# Recruitment and migration patterns reveal a key role for seed banks in the meta-population dynamics of an aquatic plant

**DOI:** 10.1038/s41598-023-37974-5

**Published:** 2023-07-12

**Authors:** Maxi Tomowski, Sissi Lozada-Gobilard, Florian Jeltsch, Ralph Tiedemann

**Affiliations:** 1 Unit of Evolutionary Biology, Institute of Biochemistry and Biology, University of Potsdam, Potsdam, Germany; 2 Plant Ecology and Nature Conservation, Institute of Biochemistry and Biology, University of Potsdam, Potsdam, Germany; 3 Biodiversity Unit, Department of Biology, University of Lund, Lund, Sweden; 4 Berlin-Brandenburg Institute of Advanced Biodiversity Research (BBIB), Berlin, Germany

**Keywords:** Molecular ecology, Population dynamics, Wetlands ecology, Population genetics, Plant ecology, Plant evolution

## Abstract

Progressive habitat fragmentation threatens plant species with narrow habitat requirements. While local environmental conditions define population growth rates and recruitment success at the patch level, dispersal is critical for population viability at the landscape scale. Identifying the dynamics of plant meta-populations is often confounded by the uncertainty about soil-stored population compartments. We combined a landscape-scale assessment of an amphibious plant’s population structure with measurements of dispersal complexity in time to track dispersal and putative shifts in functional connectivity. Using 13 microsatellite markers, we analyzed the genetic structure of extant *Oenanthe aquatica* populations and their soil seed banks in a kettle hole system to uncover hidden connectivity among populations in time and space. Considerable spatial genetic structure and isolation-by-distance suggest limited gene flow between sites. Spatial isolation and patch size showed minor effects on genetic diversity. Genetic similarity found among extant populations and their seed banks suggests increased local recruitment, despite some evidence of migration and recent colonization. Results indicate stepping-stone dispersal across adjacent populations. Among permanent and ephemeral demes the resulting meta-population demography could be determined by source-sink dynamics. Overall, these spatiotemporal connectivity patterns support mainland-island dynamics in our system, highlighting the importance of persistent seed banks as enduring sources of genetic diversity.

## Introduction

Continuous habitat has become a scarce resource—many organisms are scattered across habitat patches resulting from progressive fragmentation and degradation of natural areas^1,2^. In plants that lack long-distance dispersing propagules, landscape-scale fragmentation may limit spatial gene flow among distant patches^3–6^. Therefore, spatially isolated populations may diverge more and harbor less genetic variation, which can ultimately limit their adaptive response to selection^7,8^. However, population genetic structure may be homogenized, and local extinction risks reduced if the landscape configuration and vector availability maintain gene flow. Accordingly, effective propagule dispersal in a meta-population increases genetic connectivity which is crucial for long-term persistence. Increased immigration may particularly prevent the long-term extinction of local populations in areas subject to intensive disturbance with presumably increased mortality rates^9–11^. While the limitations of applying meta-population theory in plants have been a subject of controversy, genetic techniques have emerged as a promising tool to assess population fluctuation and migration^12–14^. However, inferring connectivity and genetic diversity from above-ground population characteristics may be misleading, as only a fraction of the actual viable population and history of local selective forces may be assessed from such single snapshot data^13,15–17^. Especially in systems with drastic short-term environmental shifts, dormancy may evolve if plants with delayed seed germination produce more surviving offspring than those whose seeds all germinate in their first year^18,19^.

Although seed banks can create a reservoir of genetic diversity^20^, rates of germination, seed mortality, and recruitment may widely differ among patches, further complicating the estimation of local extinction risks and colonization probabilities^21–23^. Tellier^24^ postulated that selection effects on plant fitness components may be amplified or mitigated by stochastic events when viable seed bank fractions are small. In the face of disturbance, increased genetic diversity can enhance the survival of subpopulations by potentially providing individuals with a wider range of adaptive capabilities. Consequently, in highly dynamic patch networks, the persistence of a plant meta-population critically depends on whether a species is equipped to maintain the exchange of genes among subdivided habitats and across successive generations^25^. Inferring current and past dispersal events can therefore unravel the dynamics of connectivity and cross-generational source-sink effects between spatially separated populations. Recent research emphasizes the role of persistent soil seed banks and their potential to decrease rates of genetic drift^24,26,27^. Moreover, under short-term environmental perturbation, seed banks enable the maintenance of potentially adaptive variation by balancing selection^24,28,29^. Indeed, soil-stored population compartments can prevent a loss of genetic diversity across multiple reproductive seasons, resulting in an overall increased effective population size^30^. This is particularly relevant in highly dynamic systems, such as arid environments, where local populations are expected to undergo strong demographic fluctuations with varying presence/absence in the above-ground population^31–34^. Numerous studies have examined the long-standing prediction of a trade-off between spatial and temporal dispersal as complementary risk spreading strategies^35–39^. While spatial dispersal is suggested to be more beneficial in spatially heterogeneous but locally stable habitats, temporal dispersal is assumed to be favored when local conditions are unpredictable.

Yet, empirical and simulation studies have discovered diverse relationships between spatial and temporal dispersal strategies, related to specific life-history traits, providing evidence for absent^40,41^, weak^32,42^, or significant^38,39^ negative covariation^36,37^. Both dispersal and dormancy have been suggested to evolve in response to the spatial and temporal heterogeneity of habitats^43–47^.

At the (meta-) population level, both seed dormancy and dispersal are predicted to increase with spatiotemporal environmental variability^43,44,48^.

Disentangling the relative prevalence of risk-spreading mechanisms in erratically fluctuating environments may also enhance the predictability of the potential for evolutionary rescue in populations under climate change. Although contributing to the resilience of fragmented populations, both dispersal strategies present distinct risks and opportunities in the context of range shifts^34^. Especially strongly structured populations that primarily track favorable conditions through recruitment from local seed rains (“dispersal in time”) are prone to extinction if the magnitude of environmental shift exceeds their niche breadth^49^. On the other hand, recurrent spatial dispersal may counteract local adaptation to conditions at the tolerance limit^50^. Consequently, climate change effects are likely to unbalance the forces of spatial and temporal rescue effects in the future^51^.

To date, only a few studies have addressed the spatiotemporal patterns of plant meta-populations since this requires long-term data on patch occupancy, a series of discrete habitat patches, and the assessment of potentially long-lived seed banks^52^. Here, we use a series of isolated wetlands as a well-suited study system to assess meta-population processes by examining above- and below-ground populations in different cohorts of the early successional short-lived monocarpic perennial species *Oenanthe aquatica* (L.) Poir^19,53^. This landscape genetic case study allows us to track signals of dispersal and thus determine the relative contribution of spatial gene flow versus seed bank recruitment in shaping local genetic variation. The target populations are distributed across small spatially distinct temporary wetlands embedded in an area of intensive agriculture. These glacially formed "kettle holes" are densely distributed habitat islands characterized by short-term changes in local hydrologic conditions, ranging from flooded to completely dry within a season, and longer-term periods of desiccation or standing water^54^. As hydrochorous pioneer *O. aquatica* is adapted to variable water levels in the study region, colonizing emerged wetland bottom when water levels recede and tolerating temporal flooding during vegetative growth. This life history likely results in population fluctuations in response to fluctuations in water levels and vegetation succession^53^. Due to the absence of connecting elements like ditches, the dispersal of seeds is likely restricted to regionally abundant animal vectors, such as deer species, wild boar, raccoons, and waterbirds^55–60^. Further, the species has been shown to form long-lived seed banks, with seed longevity, rather than dormancy, promoting storage in the soil^19,53,61,62^. We, therefore, expect the extent of past gene flow between the discrete habitat islands to be reflected in the genotype composition of the soil-stored viable seed pool^63–66^. By conducting population genetic analyses on extant populations and soil seed banks, we infer the temporal dynamics of local populations and deduce the potential connectivity among habitat islands. Our target species is a pioneer strategist with a short lifespan and our target sites and their soil strata are spatially distinct^53^. Taking advantage of these preconditions we aim to test (i) whether *O. aquatica* population structure changes over time, (ii) how the spatial configuration of habitat patches affects the genetic diversity of local populations, and (iii) if meta-population theory can explain observed population genetic patterns.

## Results

### Genetic diversity

Of the twenty patches examined, two (2a,12a) were excluded from further analyses as no *O. aquatica* individuals were found in extant or seed bank communities. Of the remaining 18 patches, six were surveyed in a previous population genetic study conducted in 2016. In two of these six patches, *O. aquatica* was absent from the standing vegetation in 2016. The 2019 survey confirmed the absence of a viable *O. aquatica* seed bank in those particular patches (Table 1). Overall 13 loci, a total of 198 alleles were scored, ranging between 6–31 alleles per locus. We detected significant deviations from Hardy–Weinberg equilibrium in at least one population at eleven out of 13 loci in each of the two extant cohorts and at eight out of 13 loci in each seed bank cohort (Supplementary Figure s1). Nevertheless, none of the loci consistently deviated from HWE proportions (as would be expected with abundant null alleles/allelic dropouts), hence all loci were included in further analyses. Low levels of Multilocus Linkage Disequilibrium r_d_ were observed in four populations of seed bank and extant cohorts ranging from 0.029 to 0.06 (Supplementary Table s1). Except for one recent seed bank cohort (P09_S1), F_IS_ values were generally positive indicating prevalent heterozygote deficiencies in most populations.

**Table 1:** Sampled *Oenanthe aquatica* populations grouped by geographic region in the *Agroscapelab* research area in northeastern Germany (see Methods), coordinates (WGS84), and sample size (n) of local populations from extant cohorts of 2019 and 2016 as well as from upper (recent; S1) and lower soil layer (historical; S2) seed banks, ‘0’ absence record of *O. aquatica,* ‘()’ recorded, but not genetically analyzed, ‘- ‘ unvisited.

Region	Location ID	Latitude	Longitude	*n*			
				2019	2016	S1	S2
Northeastern Agroscapelab	P01	53.408561	13.640170	9	0	0	0
	P02a	53.405311	13.639557	0	–	0	0
	P02	53.400064	13.671076	20	–	7	21
	P03	53.397381	13.665745	20	25	4	9
	P04	53.385815	13.699426	20	(2)	14	2
	P05	53.383518	13.708530	20	–	7	10
Central Agroscapelab	P06	53.353470	13.618264	15	24	1	15
	P07	53.352202	13.623910	18	0	0	0
	P08	53.355701	13.619981	2	–	0	0
	P09	53.345009	13.631840	20	–	9	12
	P10	53.335577	13.584429	20	–	0	0
	P11	53.342272	13.564552	20	–	11	10
	P12	53.334885	13.566890	17	–	0	0
	P13a	53.321288	13.570545	0	–	0	0
Southwestern Agroscapelab	P13	53.328058	13.524727	20	–	10	8
	P14	53.326380	13.523601	19	–	0	0
	P15	53.319181	13.539932	20	–	5	16
	P16	53.317162	13.532987	20	–	0	0
	P17	53.308486	13.552990	17	19	2	0
	P18	53.306886	13.553263	20	–	2	19

Note that sample sizes varied among soil samples due to differential germination yield.

Pairwise comparisons of the genetic diversity between extant (cohort 2019) and soil (cohort S2) populations revealed no significant differences over time in any of the parameters examined (Supplementary Table s2). A_R_ varied between 3.743 and 5.846 (mean=4.617) with consistent values among cohorts of the same population. Overall average H_E_ was 0.637 with values ranging from 0.554 to 0.702. H_O_ was generally lower than H_E_ (mean=0.571), with values ranging from 0.269 to 0.692 (Supplementary Table s1). Over the sampled cohorts, variation of population-level genetic diversity and inbreeding revealed no directional pattern.

### Spatio-temporal connectivity

**Figure 1:**
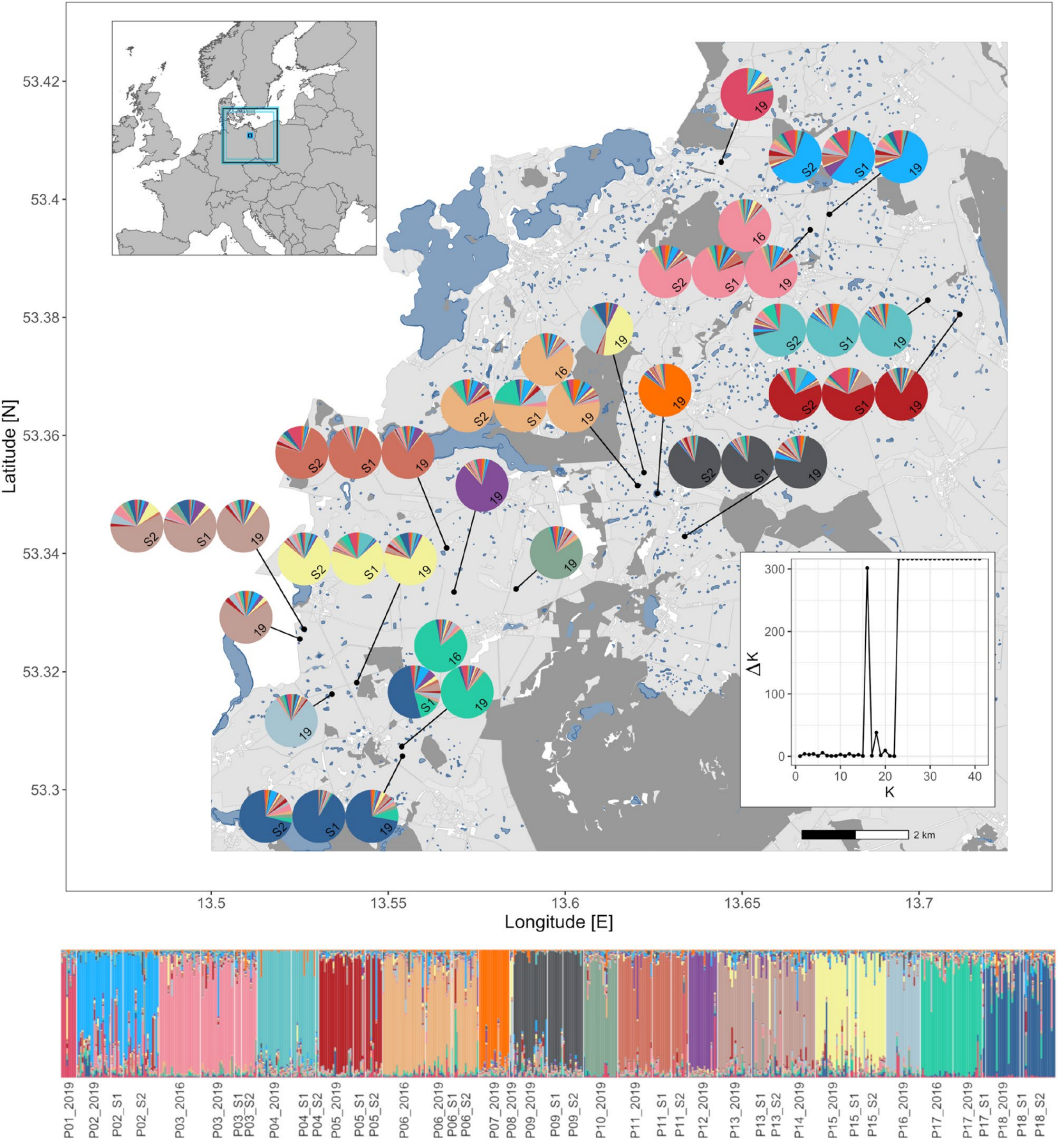
Results of STRUCTURE analysis (K = 16). *top-left*: Map extract shows Europe with sampling site indicated by a black square surrounded by a larger frame. *bottom*: Each bar represents an individual genotype with relative membership probability illustrated by distinct colours; *right*: Geographical origin of two extant and two seed bank cohorts of *Oenanthe aquatica*. Each pie-chart represents a single cohort (S1,S2: upper and lower layer seed bank, respectively; 16: 2016 above-ground and 19: 2019 above-ground annual samples) showing average relative proportion of cluster assignment revealed by STRUCTURE with optimal K = 16, illustrated by the Evanno-plot. Contiguous pies indicate cohorts of local populations. Note that not all cohorts are present in all populations. The map was generated with R version 4.2.2.^66^ using the R packages rnaturalearth^67^, sf^68^ and ggplot2^69^.

A nested Analysis of Molecular Variance (AMOVA) with cohort samples grouped according to their respective population revealed that most genetic variation (87.32%) was found within cohorts, while only 1.31% was attributable to the differentiation between cohorts within populations. Genetic differentiation among populations accounted for 11.38% of the observed genetic variance (Table 2). *STRUCTURE* analysis inferred 16 distinct genetic clusters in a pooled sample set including all cohorts under default prior settings. Individual assignment of posterior membership probabilities confirmed stable genetic composition over time, which is illustrated by the shared color.distribution in seed banks and extant cohorts within populations (Fig. 1), whereas clusters were highly segregated among sampling sites. At the same time, *STRUCTURE* indicated local gene flow as several populations showed genetic impact on neighboring populations. In one instance, two adjacent southwestern populations were assigned to the same genetic cluster (grey-brown−P13, P14). We found five kettle holes with no viable seed banks, but individuals still formed separate clusters specific to each kettle hole (Fig. 1). This suggests that recent colonization from other populations we studied is unlikely. The results based on modified cluster settings to account for unbalanced sample sizes (see methods) revealed a largely consistent population structure. However, some smaller populations without detected seed banks exhibited increased levels of admixture with neighboring sites or ambiguous cluster memberships across adjacent sites (Supplementary Figure s2) which suggests an enhanced genetic affinity between populations at the local scale, i.e., within regions.

**Table 2:** AMOVA results of spatial and temporal variation.

Source of variation	df	Sum of squares	Variance components	% variation	Fixation indices	p
Among populations	10	505.632	0.548	11.90	FCT 0.114	*p* < 0.001
Among cohorts within populations	16	98.877	0.063	1.31	FSC 0.015	*p* < 0.001
Within cohorts	861	3625.109	4.210	87.32		
Total	887	4229.618	4.822		FST 0.131	*p* < 0.001

The significance level is based on 20,000 permutations.

**Figure 2:**
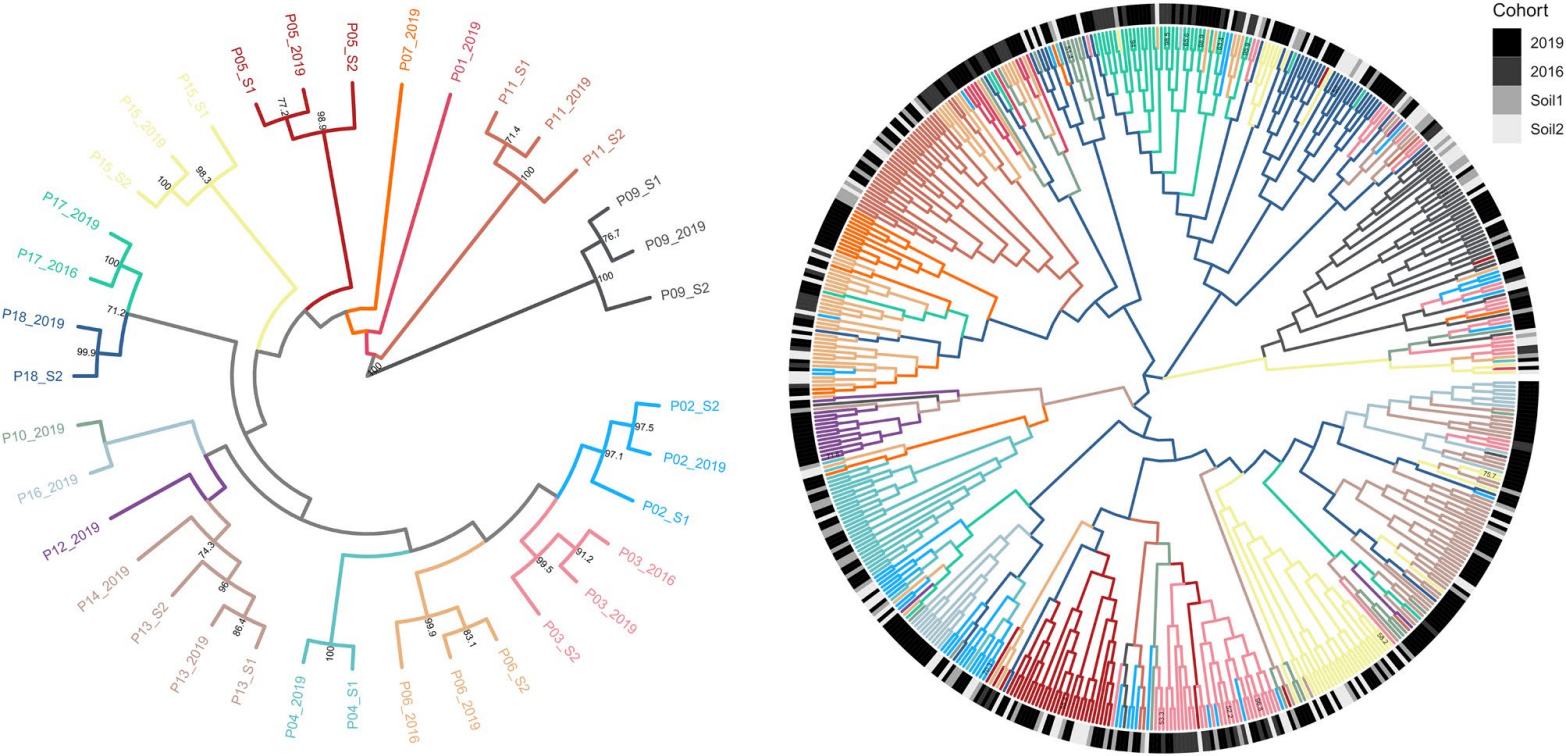
Genetic affinity of site specific cohorts Neighbor-joining trees based on Edwards’ chord distances between site-specific cohorts (left) and individuals (right) of all cohorts of *Oenanthe aquatica*. For any site, branches were colored according to the dominant genetic cluster as inferred by STRUCTURE (see Fig. 1). Numbers at branches indicate confidence values > 50% revealed by 1000 bootstrap replications. Outer bars (right) show the individuals’ cohort of origin. In most occasions, the different cohorts of a site (depicted by one specific color) cluster together.

Similar results were obtained from STRUCTURE analyses using random subsamples of genotype data with a maximum of ten samples from each site regardless of cohorts (Supplementary Figure s2). Consistent with the cluster analysis, the NJ-tree inferred from Edwards distances over all cohorts showed substantial structuring with cohorts of the same population grouped together, emphasizing the genetic affinity between cohorts indicated by bootstrap support (Fig. 2). Moreover, as revealed by STRUCTURE analysis, the populations P13 and P14 cluster together.

None of the five remaining populations with only one cohort in 2019 showed an apparent genetic affinity to any of the sampled populations. The geographical configuration of populations is not fully reflected by the tree topology, as spatially adjacent populations were not consistently grouped.

**Figure 3:**
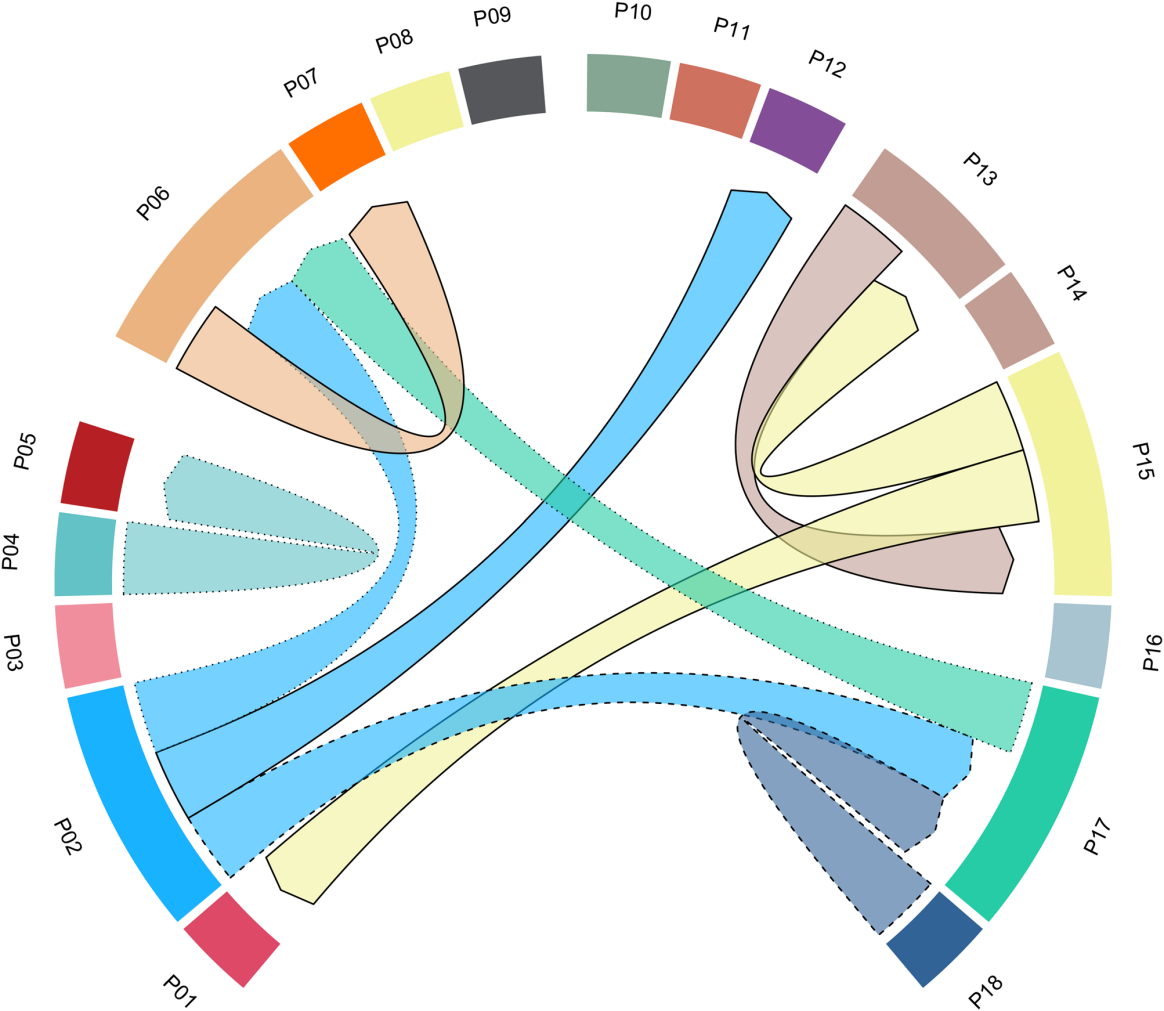
Migration pattern revealed by L_home_ Bayesian classification method in GENECLASS2 (α = 0.01). The chord diagram shows individual first-generation migrants of *Oenanthe aquatica* among sites with chords arranged clockwise in the latitudinal order of sites from North to South. Chord size is proportional to the number of migrants detected and arrows illustrate the direction of single migration events. Colors delineate dominant genetic clusters revealed by STRUCTURE analyses per site. Solid, dashed, and dotted lines depict recipient cohorts 2019, upper and lower soil seed banks, respectively.

In the individual-based NJ- tree, the overall population structure is less well resolved, but individuals are largely grouped by their population of origin without subdivision by cohort (Fig. 2), indicating low admixture across clusters. Furthermore, *GENECLASS2* determined a high self-assignment rate of 89.6%, with 512 of 570 individuals assigned to their respective sampling location. Of the 58 remaining individuals, 21 had a low probability of descent from any of the study populations, indicating either descent from unobserved genotypes or immigration from unsampled populations. 37 individuals were assigned to populations from adjacent or distant patches (Supplementary Table s3). Ten first-generation migrants (0.018%) were identified by the L_home_ approach with six of them in accordance with the assignment test (Fig. 3). Half of the recent migrations occurred between patches less than 1.5 km (160 to 1400 m) apart, while the other half were found over distances greater than 5 km (6 km to 12 km). On average, 92% of the specimens of the extant cohort 2019 descend from the previous cohorts of the same population. These results generally point towards strong among-cohort-connectivity, limited dispersal between patches, and cross-cohort gene flow. Finally, pairwise G'st comparisons between populations (pooled across cohorts) were all significant (mean = 0.330), suggesting considerable population divergence (Supplementary Table s4), indicating substantial steady spatial population structure.

Nested AMOVA analysis of pooled cohort samples, grouped by site and aggregated into three geographic regions (refer to Table 1), showed genetic variation among regions was lower (2.01%) than that among populations within regions (11.48%, Supplementary Table s5).

### Spatially explicit genetic structure

**Figure 4:**
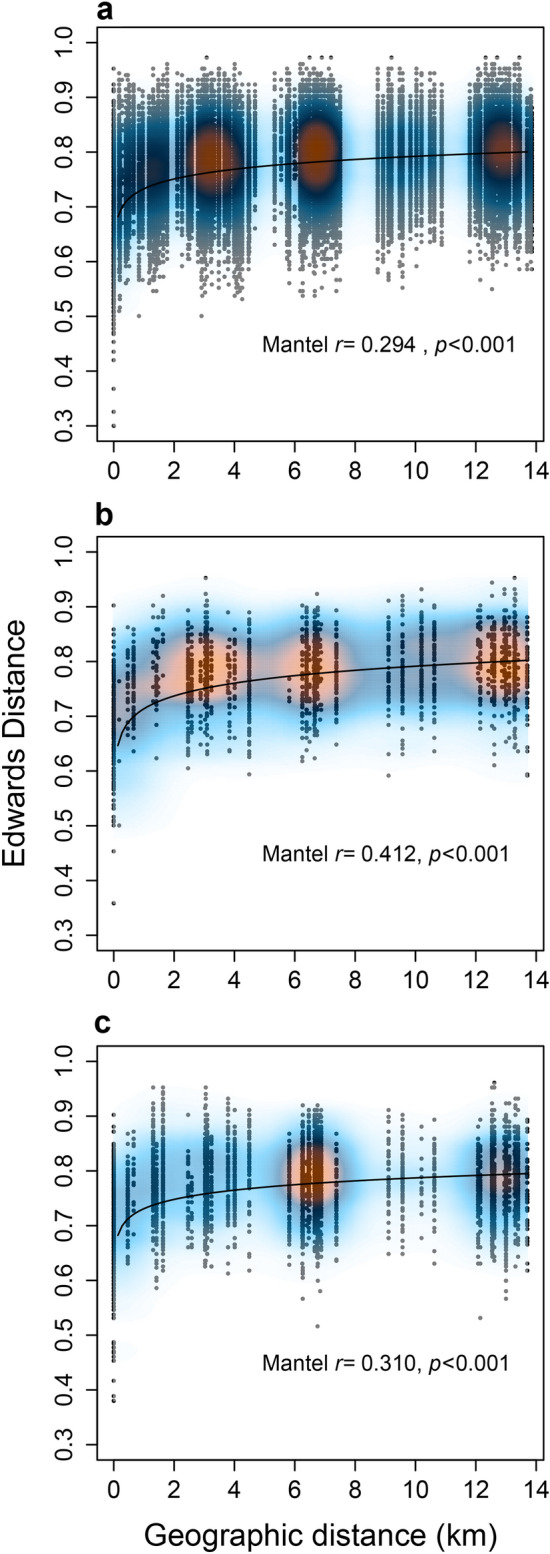
Isolation by distance (IBD) plots for cohort 2019 (**a**), upper seed bank cohorts S1 (**b**), and lower seed bank cohorts S2 (**c**) illustrate relationships between Edwards’ individual genetic distances and geographic distances among sampling sites. Colors represent the relative density of points with warmer colors indicating higher densities. The regression line and the Mantel coefficient of correlation (r) between geographic and genetic distances as well as the associated p‐value are shown for each cohort. Note that sediment genotypes are based on a lower number of cohorts which may result in lower levels of precision.

**Figure 5:**
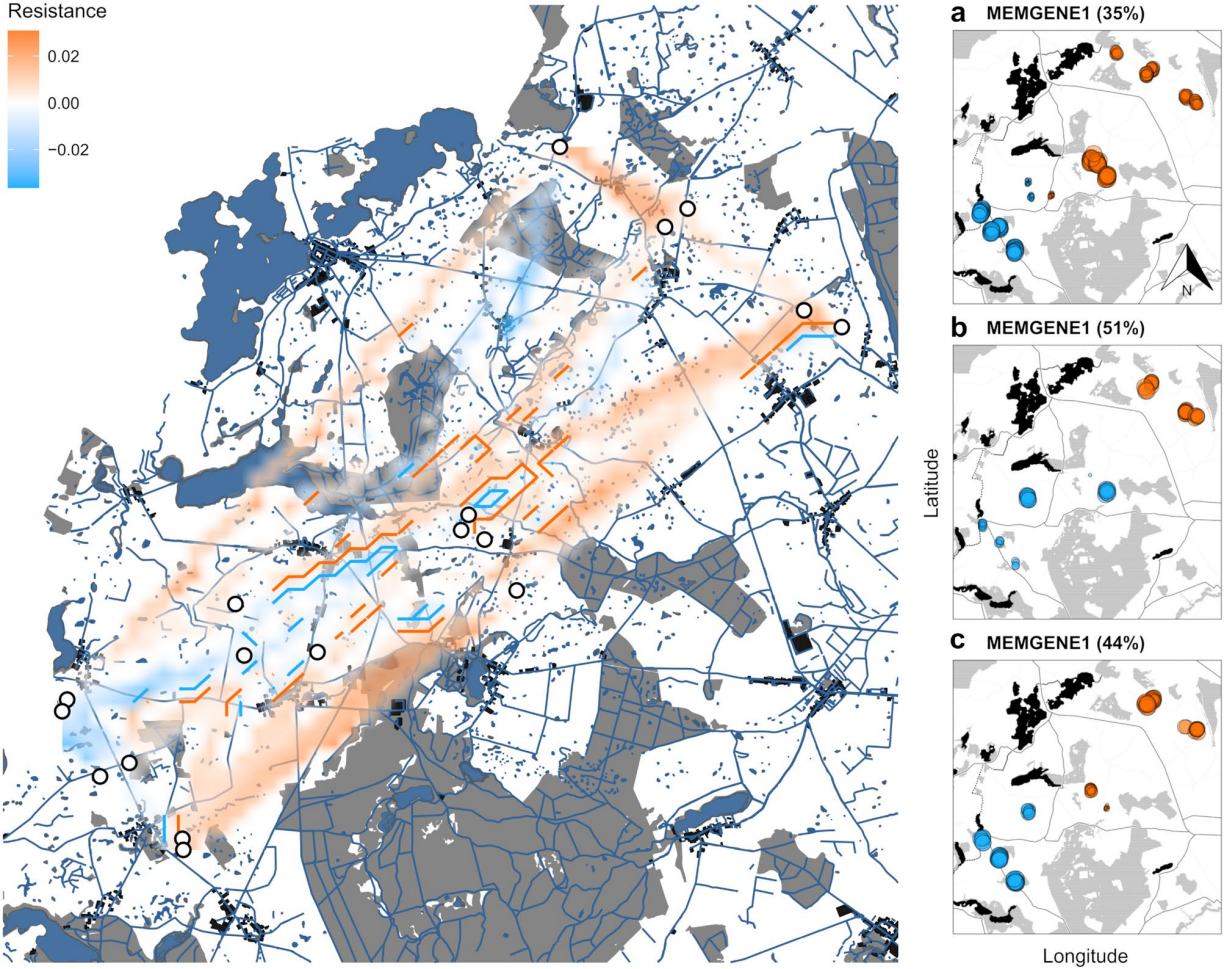
*Left:* Map displaying projected resistance values across a grid of cells, estimated by ResDisMapper using the deviation from IBD. Each individual cell (representing a 50 × 50 m area) is colored with interpolated shades of orange or bright-blue indicating high and low resistance, respectively. Contour lines delineate areas of high or low resistance with high certainty (95% CI of IBD residuals); cells lacking statistical significance are not shown. White circles are sampling sites; grey, black and dark blue areas depict woodland, urban sites and wetland, respectively. *right:* Spatial genetic structure inferred by Moran's eigenvector maps for 2019 (**a**), upper seed bank (**b**), and lower seed bank (**c**) cohorts. The graphs depict the proportion of variation explained in spatial genetic autocorrelation axes (MEMGENE1). Positive values are represented by bright blue and negative values by orange circles. Circles of similar size indicate shared genetic neighborhoods. The figure was generated with R version 4.2.2.^66^ using the R packages sf^68^, ggmap^72^ and ggplot2^69^.

A significant asymptotic increase of individual pairwise genetic distances with geographic distance was observed in the aboveground and seed bank cohorts indicating consistent spatial restriction of gene flow (Fig. 4). Accordingly, Mantel tests revealed significant isolation-by-distance patterns (*p* < 0.001) in all cohorts (r_19_ = 0.294, n_19_ = 317; r_S1_ = 0.412, n_S1_ = 72; r_S2_ = 0.3102, n_S2_ = 122). The MEM analysis showed significant but weak spatial genetic structure among populations in three analyzed cohorts (*p*_19_ < 0.001, *p*_S1_ < 0.001, *p*_S2_ = 0.019). We identified two MEMGENE axes for 2019 and three for each soil bank cohort (MEMGENE1-3) that explained 7–10% of the genetic variation underlying Moran’s eigenvector maps (MEMs; Fig. 5). Each of the first MEMGENE variables showed a similar pattern of shared genetic neighborhoods among populations explaining most of the variation (35–51%) and separating the populations into a southern and a northern subgroup. A transition zone with increased admixture along the southwestern locations is suggested for 2019 by MEMGENE1 (Fig. 5a). This is corroborated by the resistance analysis based on IBD residuals, which delineates a dispersal corridor along southwestern localities (Fig. 5). Putative dispersal barriers are inferred in areas of increased urbanization and along large forests (Fig. 5).

### Landscape configuration effects on genetic diversity

**Figure 6:**
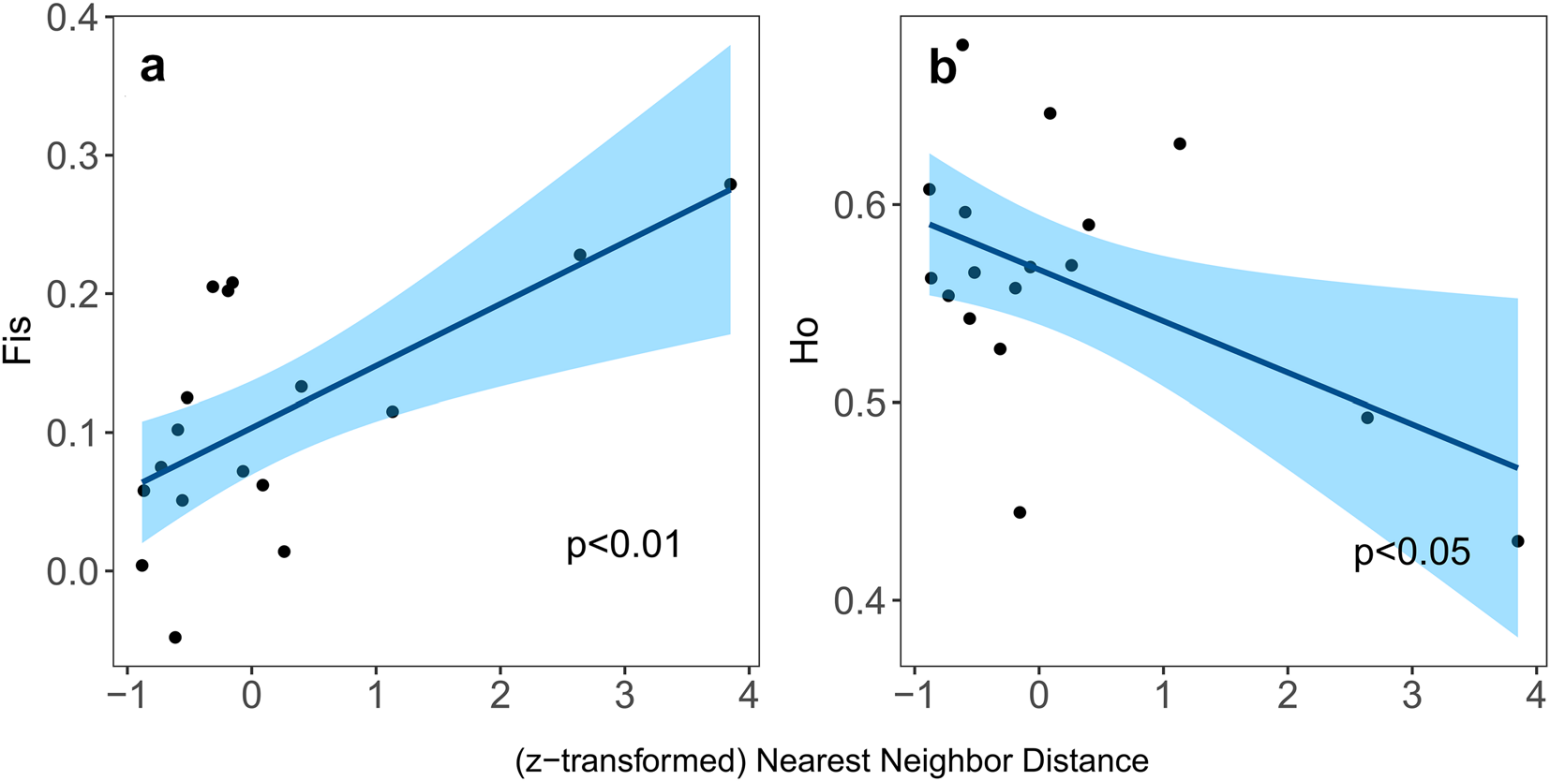
Effects of isolation on genetic diversity. GLM regressions between patch isolation measured as (z-transformed) nearest neighbor distance and (**a**) population inbreeding coefficient *Fis* (R^2^_*Pseudo*_ = 0.456;) and (**b**) observed heterozygosity *H*_*O*_ (R^2^_*Pseudo*_ = 0.252), respectively. Depicted are best-fitted GLM models based on the backward selection procedure (see Supplementary Table ss6for details)**.**

An impact of spatial isolation, i.e. nearest neighbor distance, and habitat size on measures of local genetic diversity was found in extant cohorts 2019 (Fig. 6). Increased distances to the nearest neighbor patch result in a decrease in H_O_ and increased local inbreeding. None of the genetic diversity measures were affected by the local habitat size, measured as patch area (Supplementary Table s6). Finally, neither allelic richness nor expected heterozygosity was related to any habitat configuration measure or their interaction.

## Discussion

### Spatio-temporal connectivity of O. aquatica in a dynamic landscape

Our results on spatiotemporal connectivity of *O. aquatica* across kettle holes are relevant in two major respects: firstly, we found genetic differentiation and restricted gene-flow among sampling sites in all cohorts and across varying spatial scales from a few hundred meters up to several kilometers. These findings emphasize the pronounced divergence exhibited among populations, even at small spatial scales. And secondly, the persistence of the overall connectivity patterns and the sustained levels of genetic differentiation in the study system provide robust empirical support for temporal stability in population structure. This is supported by the pronounced isolation-by-distance pattern observed in all cohorts and a clear separation of most populations (across both extant plants and seed banks) into unique clusters. In particular, long-established populations exhibit nearly unchanged allele frequencies over the period studied here. Overlapping generations in *O. aquatica* could promote cross-cohort gene flow and the maintenance of local genetic variation. Conversely, the substantial genetic divergence among populations is likely the result of genetic drift due to long-lasting habitat isolation and small population sizes. With increasing population isolation, the influence of genetic drift surpasses that of gene flow, resulting in pronounced differentiation between most of the sites. The asymptotic IBD pattern suggests that dispersal is limited to a local scale, implying that landscape barriers, i.e. the agricultural matrix, are impeding gene flow across longer distances^67^. Some admixture across sites is however suggested by the population structure analyses and inferred migration events.

STRUCTURE clustering and GENECLASS2 assignment tests showed an increase of connectivity between spatially adjacent patches in a few cases, but also indicate a (re-) colonization of patches with presumably limited or absent seed banks (i.e., those sites where no germinations were observed in any of the soil bank samples). Interestingly, of the limited number of recent migrants inferred, a considerable proportion were long-distance migrants. Animal vectors with large home ranges, such as waterbirds and large mammals may contribute to this spread by using kettle holes for shelter or foraging, facilitating seed deposition through ingestion or adhesion^55–57,59,60^. Individual sites harbor genetically distinct populations with repeated local recruitment and formation of persistent soil seed banks. Genetic variation partitioning confirmed a substantial divergence among local populations, despite occasional outcrossing with variants from nearby sites, as well as rare, but traceable, long-distance dispersal (Table 2). A small but significant proportion of genetic variation was assigned to differentiation among regions (Supplementary Table s5), underscoring genetic affinity among sites to be related to proximity.

At individual sites, genetic diversity stochastically varies among cohorts, presumably reflecting varying degrees of drift due to local environmental fluctuations (Supplementary Table s1, s2). The monocarpic life cycle of *O. aquatica*, reproducing once and then dying, may account for variations in genetic diversity and population size over time. Thus, patterns of pulsed reproduction can arise causing rapid population growth followed by a sudden decline, as new generations compete for resources. Overall, many populations in the habitat network store a long-term viable, diverse seed bank and therefore are not primarily driven by recurrent extinction/recolonization dynamics as assumed by classical meta-population theory proposed by Levins^12,68^.

However, variation in functional connectivity, including short-term changes in the occurrence of populations (as, e.g., in P01, P07) and genetically inferred dispersal across some kettle holes comprise features of meta-population dynamics. In particular, the occurrence of *O. aquatica* at patches with no detectable seed bank suggests recruitment from transient seedbanks or recent colonization.

Such colonization would require some recent synchronization of favorable conditions among donor and recipient patches^69^. Water regimes among individual kettle holes are typically asynchronous. However, short-term synchronization, e.g. intense spring flooding in a range of patches may occur occasionally. Kettle holes in this region were reported to overflow after snowmelt and to dry up during the summer period^54,70^. Stepping stone dispersal facilitated by mobile linkers, such as waterbirds^56,71^, could enable the repeated transport and accumulation of *O. aquatica* seeds among these flooded patches throughout the season. The absence of dormancy has been suggested to increase colonization success as it enables the species to rapidly exploit available resources under favorable conditions^19^. Such processes would promote regional connectivity in the long term. Further, ephemeral local populations may emerge occasionally based on randomly occurring mass effects, based e.g. on zoochorous dispersal, given that some patches may provide less suitable conditions for seed longevity^22^. Such ephemeral populations with rather transient seed banks (as represented by the grey-brown genetic cluster − P13, P14; cf. Figure 1) may emerge as a result of a 'recolonization rescue' (Hanski^72^). Short-term favorable conditions and occasional seed influx may promote the sudden emergence of large *O. aquatica* populations, capable of forming monotypic stands from a small number of individuals^53^. A community study conducted in 2015 in the same study area suggests mass effect processes to be prevalent in non-flooded patches^73^, as these may be characterized by (1) temporarily increased mobile link movements and/or (2) prolonged dry periods associated with higher seed or seedling mortality in amphibious plant species.

However, the genetic consequences of the two described dispersal scenarios will differ. High genetic diversity is expected in populations emerged from diverse migrant seed variants accumulated in short-term standing waters, e.g., at P14 and P16. Contrary, when recovering from high mortality, large populations may emerge from recent immigration of a few variants in drier environments. Here, founder effects/bottlenecks may cause reduced genetic diversity ^74^, as observed in the most spatially isolated populations, i.e., P12 and P10 (Supplementary Table s1). Such effects can be mitigated if gene flow via pollen is maintained between patches ^75^. Any of these scenarios could apply to those local populations where no soil seed banks were detected and are in line with the observed isolation-by-distance (IBD) pattern. This IBD population structure suggests a stepping-stone dynamic in our study system, which is further corroborated by contemporary dispersal being predominantly inferred among adjacent sites.

With our methodology, we cannot fully exclude that seeds in the soil remained undetected. Specifically, if a population without a detected seed bank forms a unique cluster, it could originate from scarce seeds bearing a local genotype. However, the high seed production of our studied species and our small-scale sampling of both soil and extant populations make this interpretation less probable. Therefore, we consider it more likely that those populations which form their own genetic cluster but do not hold a detectable seed bank have been colonized by specimens from a population not sampled in our study.

In future studies, it would be worth monitoring the longevity of seed banks in different hydrological regimes to identify factors that limit seed viability in soil banks. Moreover, determining population demographic structure and phenology in different environments could provide information on the role of environmental synchronization. Such information could improve the reliability of predictions about how stable the observed population connectivity may be under altered environmental conditions.

### Consequences of spatial isolation and local habitat features

Taking into account that aquatic plants cannot colonize the surrounding agriculturally utilized matrix, kettle holes play a key role as connective landscape structures with stepping-stone functions^76^, as confirmed by the inferred IBD pattern in our study system. Populations with a long-lived seed bank represent starting points for recolonization rescue, from where local variation slowly propagates across the landscape. This is in agreement with the significant latitudinal cline detected by spatially explicit analyses, separating northern and southern populations with an increased admixture at the geographical center. As precipitation is a major driver of kettle hole hydrological conditions, increased levels of connectivity between adjacent habitat patches are likely linked to local environmental synchrony^77,78^. Small-scale patch configuration effects on local diversity were already shown in plant meta-communities exhibiting a decline in species richness with increasing spatial isolation and decreasing patch area ^59,79^. We confirmed noticeable effects of isolation on local genetic diversity at the population level (Fig. 6) and found inbreeding to increase with distance from the nearest neighbor patch, which points towards the relative importance of mobile link movement ^80,81^. Limited pollinator availability in isolated patches^74^ may lead to increased levels of selfing and therefore elevated inbreeding at these sites. A reduced seed dispersal may lead to clustering of closely related offspring near parent plants which could explain enhanced inbreeding at remote habitat patches likely less frequented by large mobile linkers. The effect of patch size on local genetic diversity was negligible. Increased inter-specific competition may suppress seedling recruitment in *O. aquatica *at late successional stages when competitive species dominate. This process is likely more pronounced in larger patches ^59,60^ and ultimately results in an ephemeral population built up. Hence, unlike in populations that undergo progressive fragmentation of continuous habitat, the extent of genetic variation and connectivity in our naturally scattered study system is less a matter of patch size^79^ than a matter of small-scale environmental conditions and historical population establishment.

## Conclusion

We here assessed the spatiotemporal connectivity dynamics in a presumed aquatic plant meta-population system. Our combined approach of spatial and temporal sampling allowed us to track recent colonization events and to uncover stepping-stone dynamics with source-sink effects. These were primarily based on dispersal from local long-term persistent to spatially adjacent ephemeral populations, pointing to recolonization rescue effects as predicted by meta-population theory^82^. We showed a significant spatial genetic structure and found repeated local recruitment to largely shape the current *O. aquatica* population structure. Stepping-stone processes transgenerational gene flow are likely to maintain population viability with waves of dispersal occurring in response to temporary environmental synchrony of favorable patch conditions. Our study further highlights the crucial role of soil seed banks in serving as a source to maintain and spread variation and hence to significantly contribute to the long-term persistence of *O. aquatica* in the kettle hole system. In the face of climate change, a future drawback to population connectivity may be regional droughts that increase the distances among patches to a level that pollen and seeds cannot easily travel. A lack of immigrant variants may cause genetic erosion, further limiting the ability of local populations to adjust to harsh environmental changes^24^. It remains to be elucidated whether recurrent spatial dispersal is sufficient to maintain population connectivity under potentially increasing population separation in the course of long-term environmental change.

## Methods

### Study area

The study was conducted in the ’AgroscapeLab Quillow’ (www.zalf.de/de/struktur/eip/Seiten/AgroScapeLab.aspx), an open research platform covering an area of approximately 268 km^2^ in Brandenburg, Northeast-Germany (cf. Figure 1). The site is characterized by a mosaic of wide open areas of cropland, grassland, and mixed forest. Most agricultural land has been conventionally farmed for decades, resulting in a seasonal rotation of large monotypic stands. Several hundred small glacial wetlands (“kettle holes”) ranging in size from 0.01 to 3 ha are heterogeneously scattered throughout the area (up to 40 per km^2^)and provide unique habitat for a range of (semi-) aquatic communities^54,73,76,81,83^. Periodic changes of local water regimes vary asynchronously depending on size, steepness, and successional stage, and occur at different time scales driven by seasonal precipitation^54^.

### Study species

Fine-leaved water Dropwort, *Oenanthe aquatica* (Apiaceae) is a diploid annual to perennial monocarpic, amphibious plant species frequently occurring in disturbed eutrophic stagnant waters and ditches on muddy sediments^19,84^. As a wetland colonizer, *O. aquatica* is found in habitats with strong fluctuations in water levels. In environments with regular recurrent flooding events, populations can build up dominant stands. The species can produce high numbers of long-lived seeds, ranging from a few hundred to several tens of thousands per individual, that can be dispersed through hydrochory or animal vectors and may accumulate in the soil^19,53^. As typical in Apiacean species, flowers are protandrous and pollinated by a range of insects covering different taxonomic orders^85^. *O. aquatica* is a non-clonal, predominantly outcrossing species, however, incidences of selfing have also been observed^57^.

### Sampling and molecular analyses

Integrated with a soil community study, 20 kettle holes were selected for tissue and soil core sampling of *O. aquatica* populations in spring 2019, including two sites without *O. aquatica* in the extant plant community. These kettle holes comprised eight pairs of spatially proximate sites (several 100 meters apart) distributed across three spatial clusters (>5 km apart) as well as four intermediate sites selected as putative stepping stones between clusters (Table 1). For genotyping above-ground populations, plant material was collected from rosette leaves of juvenile plants at 18 kettle holes. Sampled individuals were separated by at least 1.5 meters. For locations with n<10 extant individuals, we sampled the whole population. As local abundances varied considerably, sample sizes ranged from two to 22 individuals (mean=15.1±7.4) per kettle hole, resulting in a total of 317 individuals. In parallel, soil cores were taken with a 15-cm drill at four 1 m^2^ squares, along a moisture gradient reflected in the extant vegetation from the ruderal edge to the central aquatic zone to account for local variation in seed bank stock at each kettle hole, as the distribution of genetic diversity in soil populations may be affected by water availability over time. Four samples were taken from each 1 m^2^ spot. Subsequently, the upper and lower 5 cm soil layers were separated, and each combined for each 1 m^2^ spot, resulting in four upper and four lower composite soil core samples per location. Core chronologies of kettle holes in the focus research region suggest that the upper soil layers reflect recent seed rains, while the lower sample seed fraction dates back several decades in the past^86^. In the greenhouse, a 2 cm thick layer (~480 ml) of each composite core sample was spread on a sandy substrate layer (2 cm ~480 ml) in a plastic tray. Using the emergence method of van der Valk & Davis (1978), a randomized flooding treatment was applied to induce germination at natural water-logged habitat conditions. This method has proven effective for assessing both persistent and transient seed banks in ephemeral wetlands^87^. Samples were exposed to natural light regime and day and night temperature of 23−30 and 18 °C, respectively. After morphological identification, seedlings other than *O. aquatica* were removed to reduce competition. Leaf tissue was sampled when reaching two-leaf-stage, yielding a total of 194 samples from viable soil populations in both layers of ten study sites, while for one further site, a viable population was found only in the upper layer. All leaf samples including soil bank seedling tissue were preserved in 96% EtOH at -20 C until further processing. Voucher specimens (species identification by Maxi Tomowski and Michael Ristow) are deposited at the Unit of Evolutionary Biology, Institute of Biochemistry and Biology, University of Potsdam, Germany under the number MT-2023-001.

Genomic DNA of *O. aquatica* populations was extracted using a modified CTAB protocol as described by Inglis et al.^88^. Each individual was genotyped for 13 polymorphic microsatellite loci described in Favre-Bac et al.^89^ (O_01, O_03, O_10, O_13, O_13, O_17, O_18, O_20, O_21, O_28, O_32, O_37, O_38, O_47). For multiplexing, forward primers of each primer pair were differently fluorescent-labeled (FAM, VIC, NED, PET) and assembled in 6 Polymerase Chain Reactions (PCR) (Supplementary Table s7). PCR products were separated via electrophoresis using a 3500 Genetic Analyser Sequencer (Applied Biosystems Hitachi). Allelic peak sizes were identified from resulting electropherograms using GeneMapper software (Version 5.0, Applied Biosystems). For three of our study sites, genotype data for the same microsatellites were available for the 2016 extant population^60^. To enable their inclusion for comparison, these data were calibrated (for laboratory methodological details, see^60^). Allele size data for a total of 579 individuals is provided in the supplementary Table s8. In subsequent analyses, we considered all emerged plants sampled within one season or seedlings emerging from one seed bank stratum as a distinct cohort, resulting in two aboveground cohorts (‘2019’, ‘2016’) and two belowground cohorts (‘Soil1’, ‘Soil2’).

### Data analyses

#### Genetic diversity and patch-network configuration

Measures of genetic diversity were estimated in R (Version 4.2.2)^90^ and R studio (Version 2022.7.1.554)^91^ for all cohorts with n ≥ 8 of each local population. For each population’s cohort and locus, we tested for significant deviation from Hardy–Weinberg-Equilibrium using the hw.test function from the *pegas* package^92^ with 1,000 permutations. To assess multilocus linkage disequilibrium (LD) we computed the standardized index of association r_d_^93^ calling the ia() function from the *poppr* package^94^. Significant deviation from the expectation of linkage equilibrium among loci was tested using 1,000 permutations, followed by a Bonferroni-correction of significance levels. We calculated descriptive statistics, including observed and expected heterozygosity (H_O_, H_E_) calling the bootstrapHet function implemented in the *popgenkit* package^95^, and rarefied allelic richness (A_R_), unbiased expected heterozygosity (_u_H_E)_, total allele number as well as the inbreeding coefficient F_IS_ using the *hierfstat* package^96^. Confidence intervals for He, Ho, and Fis were determined using 1,000 bootstrap replicates. The mlg.id function implemented in the *poppr* package^94^ was called to identify putative clones. Effects of patch area, nearest-neighbor patch distance, and their interaction on diversity estimates A_R_, H_E_, H_O_, and F_IS_ of cohorts (n ≥ 8) were assessed in R running generalized linear models (GLMs) with Gaussian distributions and z-transformed variables. We performed a stepwise backward selection to reveal best-fit models (minimum AIC) excluding variables iteratively from the full model. Effect size R^2^_pseudo_ was calculated using the *MuMin* package^97^. Patch area and distance to the nearest-neighbor patch were determined using Google Earth Pro 7.3.6.9345 (Supplementary Table s1).

#### Spatial genetic structure

Inter-individual genetic distances were calculated with the R package *poppr*^94^ using Edwards chord distance^98,99^ for seed banks and standing cohorts separately. A Mantel test for isolation by distance (IBD) was conducted to test whether genetic distances and geographic Euclidean distances are correlated, using the R package *adegenet*^100^. The significance of correlations was assessed using the mantel.randtest function with 1,000 permutations. Additionally, linear regression models were applied for each correlation by the lm function in R using log-transformed and original geographic distance data. The best-fit model based on R^2^ was chosen for IBD calculation. To identify potential barriers to gene flow and spatial genetic patterns, the mgQuick function in the MEMGENE package^100^ was used to integrate geographic configuration with genetic distance based on Moran’s eigenvector maps (MEM)^101^. This stepwise analysis was performed separately for each set of cohorts using Edwards genetic distances between individuals and patch coordinates. Significant MEM eigenvectors were obtained through forward selection with 1,000 permutations followed by a redundancy analysis (RDA) to determine the proportion of explained variation by each eigenvector. Finally, MEMGENE axes were computed to assess spatial genetic neighborhoods. Moreover, we conducted a resistance analysis based on the deviation from IBD for the aboveground population 2019 to identify recent dispersal corridors and barriers, using the *ResDisMapper* package^102^. In this analysis, the residuals obtained from the isolation by distance method (see above) were considered as line segments connecting each pair of individuals across our target landscape. To calculate resistance, the landscape was divided into a grid of cells and the resistance of each cell was computed by averaging the IBD residuals of all lines that traverse the cell. The significance of resistance was obtained using 1,000 permutations. We mapped interpolated resistance scores as well as corridors and barriers based on confidence limits. This method requires no prior information about dispersal-limiting environmental characteristics and has been shown to produce accurate resistance values at small spatial scales^102,103^.

#### Spatio-temporal genetic variation

To test whether individuals can be assigned to distinct genetic groups following increased differentiation among sampled locations, Bayesian genetic clustering was performed using *STRUCTURE* Version 2.3.4^104^. Using a fused dataset comprising samples of all cohorts, the likelihood for the number of putative genetic clusters (K) was estimated to evaluate structuring among years and sites. We ran ten simulations for each K from two to 42 (the total number of local cohorts across all populations) under the admixture model without prior population information. Parameter settings involved a burn-in period of 100,000 iterations followed by 500,000 Markov Chain Monte Carlo (MCMC) repetitions. The optimal number of clusters was determined by plotting the likelihood of K for each value of K (*LnPr*⌈*X*|*K*⌉) using the △*K* method^105^ in the *Pophelper* package^106^ in R. Clustering results of individual membership probabilities were visualized with the implemented *pophelperShiny* App.

The relative contribution of each inferred genetic cluster per population and cohort was illustrated as a pie chart on a physical map. To account for the putative effects of differences in sample size in STRUCTURE, we conducted a separate analysis using the admixture model with uncorrelated allele frequencies and an ancestry prior α = 0.026 (1/number of expected populations^107^) using the same settings as in the previous analyses. Additional STRUCTURE analyses with a maximum of ten random subsamples from each site, regardless of cohorts, were performed for ten compiled sample sets based on the default model priors and the same settings. A complementary neighbor-joining tree (NJ- tree) based on Edwards genetic distance with 1,000 bootstrap replicates was constructed in *ape* for cohort level (n ≥ 5) and individual level analyses to assess spatial and temporal divergence simultaneously NJ-trees were visualized using *ggtree*^108^.

To test for partitioning of variation among populations and between cohorts, an analysis of molecular variance (AMOVA) was performed in *Arlequin* Version 3.5^109^ using 20,000 permutations. We, therefore, selected a subset of populations, for which at least two of four cohorts (2019, 2016, S1, S2) were available with a minimum sample size of n = 8 per cohort. Levels of spatial population structuring were further examined using pairwise G’st^110^ among populations. Given the low variability across cohorts within populations (see Results), cohorts (n ≥ 8) were pooled for any population before the calculation of G’st. We tested for the significance of G’st values by estimating 95% confidence intervals based on 1,000 bootstraps over loci using the *diveRsity* package^111^. We performed a further nested AMOVA analysis on pooled cohort samples grouped by each site (n_pooled_ > 8) and aggregated into three regions (Table 1) to examine the partitioning of variance within and among regions.

Recent dispersal and signals of immigration were inferred by a stepwise assignment procedure in *GENECLASS2*^112^. First, we conducted the implemented self-assignment test to assign or exclude reference populations as possible origins for each individual. Therefore, we calculated individual assignment probabilities by the Monte Carlo resampling method proposed by Paetkau et al. (2004)^113^ with 10,000 simulations and assessed source population log-likelihood under the Bayesian Criterion of Rannala & Mountain (1997)^114^. As a baseline for individual assignments, we pooled the data from all cohorts per population^115^. A probability threshold of 0.01 was set to exclude individuals likely originating from unsampled sources. Additionally, we detected potential first-generation migrants under the Bayesian Criterion of Rannala & Mountain^114^ using the same resampling algorithm to calculate the likelihood of individuals belonging to their local gene pool or external ones. A probability threshold of α = 0.01 was set to reduce the probability of false positives (i.e., incorrect identification of residents as immigrants) ^114,116^. We further examined dispersal in time by determining the proportion of the most recent cohorts (2019) classified as descendants of earlier cohorts. For this, we created representative reference data for each local population by removing putative immigrant samples detected during the first two steps^115^ resulting in ten local datasets. 2019 population data and reference data from previous cohorts (2016, S1, S2; only cohorts with n ≥ 5 considered) were selected to perform an assignment test as earlier (α = 0.01).

### Declaration on ethics and research permits

We hereby declare that our experimental research and field studies on cultivated and wild plants, including the collection of plant material, are in accordance with relevant institutional, national, and international guidelines and legislation. Leaf samples of *Oenanthe aquatica* were collected under a sampling permit issued by the State environmental authority LfU Brandenburg (Germany).

## Supplementary Information

Supplementary Information 1.

Supplementary Information 2.

## Data Availability

All datasets generated and analyzed during this study are available in the main text and supplementary material. Additional information can be obtained from the corresponding author on request.
