# Supplementary material for: Recruitment and migration patterns reveal a key role for seed banks in the meta-population dynamics of an aquatic plant
Source: Sci Rep. 2023 Jul 12;13:11269. doi: 10.1038/s41598-023-37974-5 (PMC10338435; doi:10.1038/s41598-023-37974-5)
Supplement: Supplementary file 1 — Supplementary Information 1. [file 41598_2023_37974_MOESM1_ESM.docx]

*Supplementary Material*

**Recruitment and migration patterns reveal a key role for seed banks in the meta-population dynamics of an aquatic plant**

# Tomowski M., Lozada-Gobilard S., Jeltsch F., Tiedemann R.

*Results*

**Table s1** Genetic diversity measures and location information. Sample size (n), observed and expected heterozygosity (Ho,He, with 95% confidence limits), unbiased He (_u_He), allelic richness (Ar), number of alleles (n.all), and inbreeding coefficient (Fis, with 95% confidence limits) as well as multilocus linkage disequilibrium (rd) are listed for each cohorts sample of a population (Loc Cohort). Patch size (Area) and distance to nearest-neighbor patch (nN_dist) are given for each location (Loc ID)

.

| ***Loc***  ***ID_ZALF_*** | ***Loc***  ***Cohort*** | ***Loc ID*** | ***n*** | ***rd*** | ***p_rd*** | ***Cohort*** | ***n.all*** | ***Ar*** | ***Ho (C_l_-C_u_)*** | ***He (C_l_-C_u_)*** | ***_u_He*** | ***Fis (C_l_-C_u_)*** | ***Area***  ***[m^2^]*** | ***nN_dist***  ***[m]*** |
| --- | --- | --- | --- | --- | --- | --- | --- | --- | --- | --- | --- | --- | --- | --- |
| 911 | P01_2019 | P01 | 9 | 0.038 | 1 | 2019 | 53 | 3.903 | 0.444 (0,325-0.547) | 0.540 (0.404-0.580) | 0.580 | 0.208 (0.094-0.372) | 1,876.86 | 156.00 |
| 814 | P02_2019 | P02 | 20 | -0.021 | 1 | 2019 | 73 | 4.567 | 0.631 (0,577-0.688) | 0.683 (0.636-0.687) | 0.702 | 0.115 (-0.005-0.214) | 400.60 | 296.44 |
| 814 | P02_S1 | P02 | 7 | - | - | Soil1 | - | - | - | - | - | - |  |  |
| 814 | P02_S2 | P02 | 21 | -0.003 | 1 | Soil2 | 78 | 4.509 | 0.607 (0,544-0.668) | 0.657 (0.609-0.664) | 0.675 | 0.079 (0.014-0.174) |  |  |
| 807 | P03_2019 | P03 | 20 | 0.022 | 0.526 | 2019 | 64 | 4.13 | 0.527 (0.451-0.604 | 0.644 (0.584-0.655) | 0.664 | 0.205 (0.123-0.296) | 2,401.11 | 138.73 |
| 807 | P03_S1 | P03 | 4 | - | - | Soil1 | - | - | - | - | - | - |  |  |
| 807 | P03_S2 | P03 | 9 | -0.011 | 1 | Soil2 | 60 | 4.475 | 0.641(0.567-0.718 | 0.623 (0.539-0.620) | 0.662 | 0.009 (-0.088-0.134) |  |  |
| 807 | P03_2016 | P03 | 25 | 0.029 | 0.03 | 2016 | 66 | 4.018 | 0.599 (0.542-0.656 | 0.643 (0.598-0.657) | 0.658 | 0.085 (-0.012-0.189) |  |  |
| 287 | P04_2019 | P04 | 20 | 0.006 | 1 | 2019 | 78 | 4.644 | 0.646 (0.588-0.769) | 0.664 (0.611-0.676) | 0.682 | 0.062 (-0.039-0.152) | 3,942.56 | 182.57 |
| 287 | P04_S1 | P04 | 14 | -0.009 | 1 | Soil1 | 69 | 4.527 | 0.576 (0.513-0.634) | 0.638 (0.571-0.645) | 0.665 | 0.148 (0.052-0.217) |  |  |
| 287 | P04_S2 | P04 | 2 | - | - | Soil2 | - | - | - | - | - | - |  |  |
| 260 | P05_2019 | P05 | 20 | 0.004 | 1 | 2019 | 68 | 4.141 | 0.596 (0.551-0.642) | 0.649 (0.606-0.654) | 0.667 | 0.102 (0.028-0.192) | 522.21 | 108.22 |
| 260 | P05_S1 | P05 | 7 | -0.037 | 1 | Soil1 | - | - | - | - | - | - |  |  |
| 260 | P05_S2 | P05 | 10 | 0.06 | 0.045 | Soil2 | 58 | 4.248 | 0.589 (0.466-0.700) | 0.638 (0.541-0.644) | 0.677 | 0.087 (-0.027-0.295) |  |  |
| 1189 | P06_2019 | P06 | 15 | 0.026 | 0.832 | 2019 | 68 | 4.43 | 0.590 (0.503-0.682) | 0.650 (0.584-0.660) | 0.675 | 0.133 (0.022-0.257) | 1,849.69 | 216.47 |
| 1189 | P06_S1 | P06 | 1 | - | - | Soil1 | - | - | - | - | - | - |  |  |
| 1189 | P06_S2 | P06 | 15 | 0.009 | 1 | Soil2 | 71 | 4.623 | 0.585 (0.528-0.641) | 0.658 (0.586-0.672) | 0.684 | 0.158 (0.048-0.259) |  |  |
| 1189 | P06_2016 | P06 | 24 | 0.01 | 1 | 2016 | 75 | 4.314 | 0.644 (0.599-0.694) | 0.640 (0.601-0.649) | 0.651 | 0.018 (-0.047-0.074) |  |  |
| 2484 | P07_2019 | P07 | 18 | -0.001 | 1 | 2019 | 57 | 3.771 | 0.568 (0.489-0.641) | 0.578 (0.530-0.588) | 0.595 | 0.072 (-0.021-0.122) | 11,266.48 | 165.24 |
| 2489 | P08_2019 | P08 | 2 | - | - | 2019 | - | - | - | - | - | - | 1,341.99 | 272.73 |
| 599 | P09_2019 | P09 | 20 | 0.003 | 1 | 2019 | 71 | 4.299 | 0.608 (0.550-0.658 | 0.599 (0.553-0.607) | 0.615 | 0.004 (-0.097-0.124) | 1,169.28 | 76.66 |
| 599 | P09_S1 | P09 | 9 | 0.041 | 0.5 | Soil1 | 52 | 3.867 | 0.581 (0.461-0.692) | 0.547 (0.427-0.573) | 0.579 | -0.028 (-0.121-0.082) |  |  |
| 599 | P09_S2 | P09 | 12 | 0.038 | 0.324 | Soil2 | 59 | 3.993 | 0.484 (0.404-0.569) | 0.527 (0.426-0.558) | 0.554 | 0.109 (-0.010-0.236) |  |  |
| 1165 | P10_2019 | P10 | 20 | 0.004 | 1 | 2019 | 61 | 3.825 | 0.492 (0.423-0.556) | 0.608 (0.555-0.619) | 0.627 | 0.228 (0.076-0.379) | 3,755.79 | 461.10 |
| 2544 | P11_2019 | P11 | 20 | -0.017 | 1 | 2019 | 62 | 3.743 | 0.542 (0.481-0.608) | 0.567 (0.520-0.576) | 0.582 | 0.051 (-0.030-0.173) | 756.33 | 111.99 |
| 2544 | P11_S1 | P11 | 11 | -0.002 | 1 | Soil1 | 51 | 3.643 | 0.545 (0.475-0.627) | 0.548 (0.473-0.566) | 0.575 | 0.045 (-0.080-0.211) |  |  |
| 2544 | P11_S2 | P11 | 10 | -0.008 | 1 | Soil2 | 54 | 4.822 | 0.524 (0.472-0.600) | 0.592 (0.503-0.604) | 0.634 | 0.118 (-0.014-0.341) |  |  |
| 2547 | P12_2019 | P12 | 17 | 0.058 | 0.017 | 2019 | 50 | 3.392 | 0.430 (0.348-0.511) | 0.549 (0.480-0.580) | 0.570 | 0.279 (0.102-0.340) | 2,865.01 | 593.00 |
| 12 | P13_2019 | P13 | 20 | -0.008 | - | 2019 | 70 | 4.087 | 0.569 (0.519-0.615) | 0.562 (0.512-0.576) | 0.577 | 0.014 (-0.066-0.100) | 9,000.60 | 201.36 |
| 12 | P13_S1 | P13 | 10 | -0.013 | 1 | Soil1 | 53 | 3.891 | 0.562 (0.485-0.631) | 0.597 (0.516-0.600) | 0.632 | 0.104 (-0.007-0.216) |  |  |
| 12 | P13_S2 | P13 | 8 | -0.044 | 1 | Soil2 | 47 | 5.846 | 0.490 (0.317-0.644) | 0.549 (0.405-0.570) | 0.598 | 0.152 (0.026-0.321) |  |  |
| 13 | P14_2019 | P14 | 19 | -0.004 | 1 | 2019 | 69 | 4.169 | 0.563 (0.498-0.632) | 0.594 (0.538-0.608) | 0..612 | 0.058 (0.005-0.148) | 273.95 | 78.21 |
| 37 | P15_2019 | P15 | 20 | 0.035 | 0.085 | 2019 | 83 | 4.931 | 0.681 (0.624-0.731) | 0.638 (0.575-0.659) | 0.653 | -0.048 (-0.076-0.008) | 848.98 | 105.76 |
| 37 | P15_S1 | P15 | 5 | - | - | Soil1 | - | - | - | - | - | - |  |  |
| 37 | P15_S2 | P15 | 16 | 0.009 | 1 | Soil2 | 73 | 4.58 | 0.631 (0.593-0.677) | 0.626 (0.573-0.638) | 0.651 | 0.016 (-0.076-0.126) |  |  |
| 36 | P16_2019 | P16 | 20 | -0.005 | 1 | 2019 | 65 | 3.937 | 0.554 (0.481-0.623) | 0.583 (0.519-0.606) | 0.599 | 0.075 (-0.012-0.192) | 2,315.28 | 93.37 |
| 1598 | P17_2019 | P17 | 17 | 0.033 | 0.221 | 2019 | 67 | 4.293 | 0.566 (0.489-0.638) | 0.620 (0.555-0.632) | 0.641 | 0.125 (0.029-0.234) | 2,905.34 | 116.2 |
| 1598 | P17_2016 | P17 | 19 | 0.043 | 0.01 | 2016 | 64 | 4.219 | 0.555 (0.502-0.607) | 0.625 (0.569-0.637) | 0.645 | 0.134 (0.042-0.232) |  |  |
| 1598 | P17_S1 | P17 | 2 | - | - | Soil1 | - | - | - | - | - | - |  |  |
| 1597 | P18_2019 | P18 | 20 | 0.014 | 1 | 2019 | 65 | 4.147 | 0.558 (0.477-0.638) | 0.677 (0.623-0.683) | 0.698 | 0.202 (0.096-0.308) | 743.10 | 152.08 |
| 1597 | P18_S2 | P18 | 19 | 0.024 | 1 | Soil2 | 65 | 4.16 | 0.611 (0.530-0.680) | 0.645 (0.596-0.653) | 0.664 | 0.068 (-0.029-0.186) |  |  |
| 1597 | P18_S1 | P18 | 2 | - | - | Soil1 | - | - | - | - | - | - |  |  |

**Figure s1** Heat maps showing significant departures from Hardy-Weinberg-Equilibrium (HWE) with single loci in columns and cohorts of local populations denoted as ‘19’ (2019), ‘16’ (2016), ’S1’ and ’S2’ in rows; grey indicates loci presumed of not being in HWE with p ≤ 0.05 in the indicated cohort


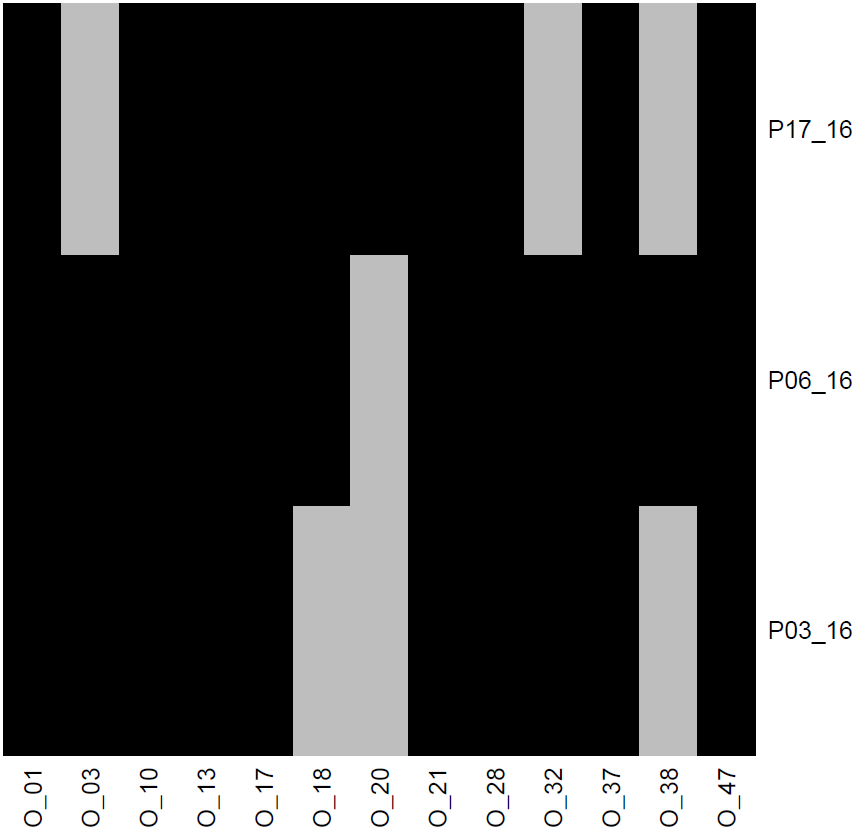

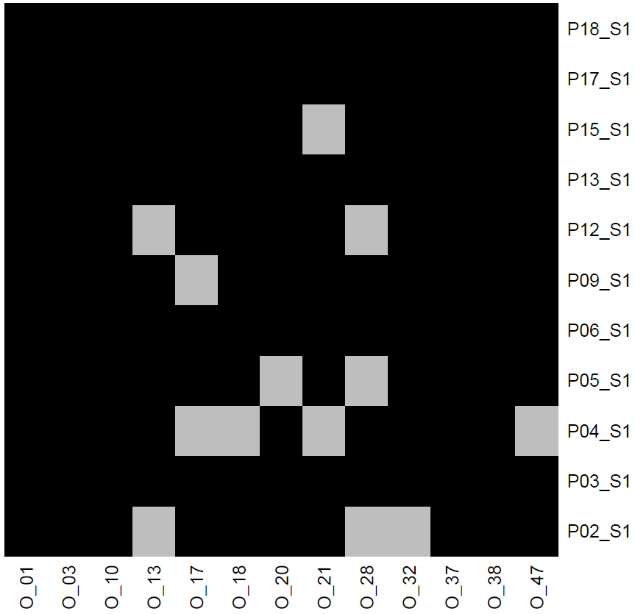

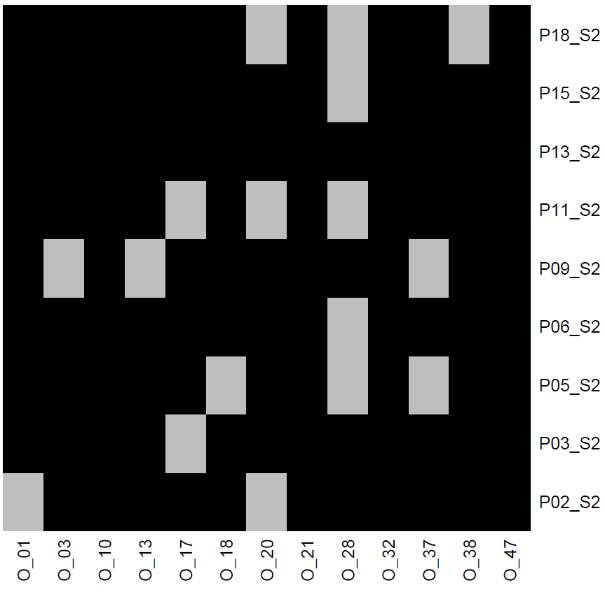

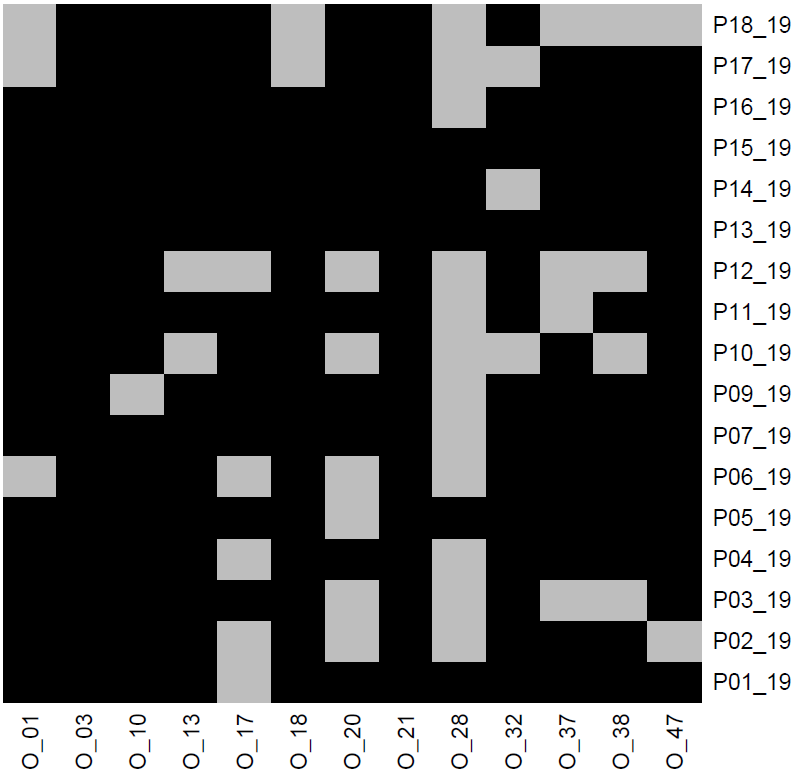


**Table s2** Results of the paired Wilcoxon test for the genetic diversity parameters expected heterozygosity (He), observed heterozygosity (Ho), allelic richness (Ar), and Inbreeding coefficient (Fis) among extant (cohort 2019) and soil (cohort Soil 2) populations of Oenanthe aquatica.

| Wilcoxon paired | |  |  |  |  |  |  |
| --- | --- | --- | --- | --- | --- | --- | --- |
|  | ***group1*** | ***group2*** | ***n1*** | ***n2*** | ***statistic*** | ***p*** | ***p signif*** |
| **He** |  |  |  |  |  |  |  |
| value | Cohort 2019 | Cohort S2 | 9 | 9 | 25 | 0.820 | ns |
| **Ho** |  |  |  |  |  |  |  |
| value | Cohort 2019 | Cohort S2 | 9 | 9 | 31 | 0.359 | ns |
| **Ar** |  |  |  |  |  |  |  |
| value | Cohort 2019 | Cohort S2 | 9 | 9 | 14 | 0.359 | ns |
| **Fis** |  |  |  |  |  |  |  |
| value | Cohort 2019 | Cohort S2 | 9 | 9 | 20 | 0.820 | ns |

**
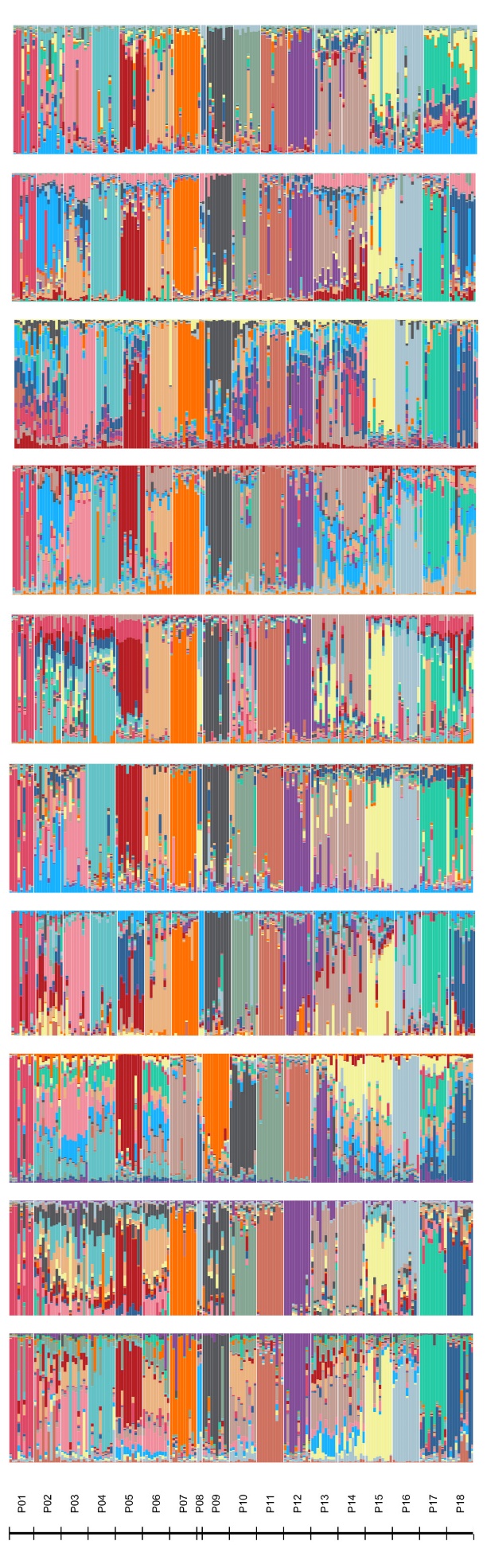
**
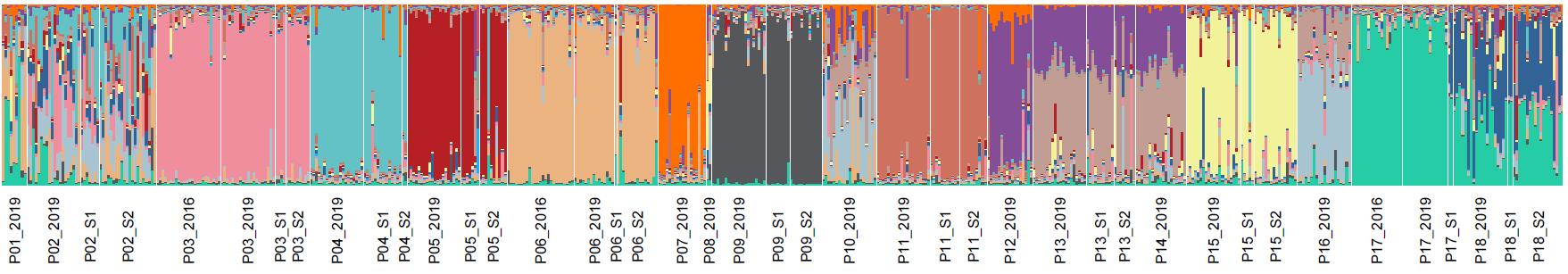


**Figure s2** Results of complementary STRUCTURE analyses (K=16). left: Each bar chart represents the average of ten STRUCTURE runs conducted using random sample sets (n≤10) per site regardless of cohort. Single bars represent average relative membership probability of individual genotypes illustrated by distinct colors. right: Bar chart illustrating average relative membership probability using the alternative model prior alpha=0.026 and the uncorrelated allele frequency model in STRUCTURE analysis including the entire sample set.

**Table s3** Results of self-assignment test conducted in GENECLASS2. Shown are individuals with a high probability to belong to a population other than their sampling population.

| ***Individual ID*** | ***Sampled Population*** | ***Reference Population*** |
| --- | --- | --- |
| 911_9 | P01 | P02 |
| 911_6 | P01 | P02 |
| 814_4u_6 | P02 | P05 |
| 814_4u_2 | P02 | P01 |
| 814_3_3o | P02 | P01 |
| 807_26 | P03 | P02 |
| 807_22 | P03 | P05 |
| 807_1_1o | P03 | P02 |
| 260_4o_2a | P05 | P02 |
| 260_3u_3 | P05 | P04 |
| 260_1_3u | P05 | P02 |
| 1189_9_3u | P06 | P02 |
| 2484_7 | P07 | P06 |
| 2484_3 | P07 | P06 |
| 2489_2 | P08 | P15 |
| 599_9 | P09 | P02 |
| 1165_29 | P10 | P06 |
| 1165_14 | P10 | P15 |
| 1165_11 | P10 | P15 |
| 2544_8 | P11 | P02 |
| 2544_4_4u | P11 | P01 |
| 2544_18 | P11 | P18 |
| 2544_16 | P11 | P02 |
| 2547_7 | P12 | P02 |
| 2547_3 | P12 | P02 |
| 12_7_4o | P13 | P18 |
| 12_4u_4 | P13 | P05 |
| 12_3_4u | P13 | P15 |
| 12_2 | P13 | P02 |
| 12_18 | P13 | P15 |
| 12_17 | P13 | P15 |
| 13_18 | P14 | P06 |
| 36_6 | P16 | P02 |
| 36_21 | P16 | P02 |
| 1598_1_4o | P17 | P18 |
| 1598_1_2o | P17 | P02 |
| 1597_3 | P18 | P17 |
| 1597_2_3u | P18 | P04 |
| 1597_15 | P18 | P17 |
| 1597_1_3u | P18 | P02 |

**Table s4** Pairwise values of genetic divergence (Hedrick’s G’st, 2005) as well as upper and lower confidence limits calculated among single populations (pooled across cohorts). ‘BC’ is indicative for bias corrected estimates.

| **comparison** | | | **Mean G’st** | **BC**  **Mean G’st** | **Lower**  **95%CI** | **Upper**  **95%CI** | **BC Lower**  **95%CI** | **BC Upper 95%CI** |
| --- | --- | --- | --- | --- | --- | --- | --- | --- |
| **P01** | **vs.** | **P02** | 0.3509 | **0.2989** | 0.2706 | 0.4385 | 0.2186 | 0.3864 |
| **P01** | **vs.** | **P03** | 0.4291 | **0.3869** | 0.358 | 0.5106 | 0.3158 | 0.4684 |
| **P01** | **vs.** | **P04** | 0.4702 | **0.4268** | 0.3788 | 0.5566 | 0.3354 | 0.5132 |
| **P01** | **vs.** | **P05** | 0.4065 | **0.3555** | 0.3171 | 0.5124 | 0.2661 | 0.4614 |
| **P01** | **vs.** | **P06** | 0.2882 | **0.2359** | 0.2136 | 0.3784 | 0.1613 | 0.3261 |
| **P01** | **vs.** | **P07** | 0.4188 | **0.3668** | 0.3359 | 0.5147 | 0.2839 | 0.4627 |
| **P01** | **vs.** | **P09** | 0.4925 | **0.4604** | 0.4004 | 0.599 | 0.3683 | 0.567 |
| **P01** | **vs.** | **P10** | 0.3551 | **0.296** | 0.2671 | 0.4524 | 0.208 | 0.3933 |
| **P01** | **vs.** | **P11** | 0.3709 | **0.3238** | 0.3032 | 0.456 | 0.2561 | 0.4089 |
| **P01** | **vs.** | **P12** | 0.4759 | **0.4284** | 0.398 | 0.5679 | 0.3505 | 0.5204 |
| **P01** | **vs.** | **P13** | 0.4168 | **0.3744** | 0.3311 | 0.5106 | 0.2887 | 0.4682 |
| **P01** | **vs.** | **P14** | 0.5321 | **0.4901** | 0.4543 | 0.623 | 0.4123 | 0.5811 |
| **P01** | **vs.** | **P15** | 0.455 | **0.4148** | 0.3661 | 0.5497 | 0.3259 | 0.5095 |
| **P01** | **vs.** | **P16** | 0.5132 | **0.4711** | 0.4349 | 0.6086 | 0.3927 | 0.5664 |
| **P01** | **vs.** | **P17** | 0.2982 | **0.241** | 0.2329 | 0.3823 | 0.1757 | 0.325 |
| **P01** | **vs.** | **P18** | 0.4012 | **0.3518** | 0.3012 | 0.5105 | 0.2518 | 0.4612 |
| **P02** | **vs.** | **P03** | 0.1856 | **0.1595** | 0.1488 | 0.2216 | 0.1227 | 0.1955 |
| **P02** | **vs.** | **P04** | 0.2014 | **0.1697** | 0.1627 | 0.2428 | 0.131 | 0.2111 |
| **P02** | **vs.** | **P05** | 0.262 | **0.2302** | 0.2073 | 0.3176 | 0.1755 | 0.2858 |
| **P02** | **vs.** | **P06** | 0.1956 | **0.1725** | 0.1552 | 0.2393 | 0.132 | 0.2162 |
| **P02** | **vs.** | **P07** | 0.3319 | **0.3014** | 0.2692 | 0.3925 | 0.2388 | 0.362 |
| **P02** | **vs.** | **P09** | 0.3388 | **0.3197** | 0.2893 | 0.3865 | 0.2702 | 0.3674 |
| **P02** | **vs.** | **P10** | 0.2846 | **0.2484** | 0.2234 | 0.3535 | 0.1872 | 0.3173 |
| **P02** | **vs.** | **P11** | 0.3207 | **0.2993** | 0.2708 | 0.3756 | 0.2494 | 0.3542 |
| **P02** | **vs.** | **P12** | 0.4183 | **0.391** | 0.3403 | 0.4955 | 0.313 | 0.4682 |
| **P02** | **vs.** | **P13** | 0.3709 | **0.3507** | 0.3096 | 0.4326 | 0.2893 | 0.4124 |
| **P02** | **vs.** | **P14** | 0.3813 | **0.352** | 0.3198 | 0.45 | 0.2904 | 0.4206 |
| **P02** | **vs.** | **P15** | 0.3505 | **0.3288** | 0.2924 | 0.4154 | 0.2707 | 0.3937 |
| **P02** | **vs.** | **P16** | 0.3237 | **0.2932** | 0.2672 | 0.385 | 0.2367 | 0.3546 |
| **P02** | **vs.** | **P17** | 0.3462 | **0.3234** | 0.2966 | 0.3995 | 0.2738 | 0.3768 |
| **P02** | **vs.** | **P18** | 0.2623 | **0.2344** | 0.2111 | 0.3187 | 0.1832 | 0.2908 |
| **P03** | **vs.** | **P04** | 0.2625 | **0.2365** | 0.2223 | 0.3121 | 0.1962 | 0.286 |
| **P03** | **vs.** | **P05** | 0.2936 | **0.2661** | 0.2398 | 0.3481 | 0.2122 | 0.3206 |
| **P03** | **vs.** | **P06** | 0.2358 | **0.2159** | 0.1942 | 0.2808 | 0.1743 | 0.2609 |
| **P03** | **vs.** | **P07** | 0.3866 | **0.3608** | 0.3301 | 0.446 | 0.3043 | 0.4201 |
| **P03** | **vs.** | **P09** | 0.2994 | **0.2824** | 0.2601 | 0.347 | 0.243 | 0.33 |
| **P03** | **vs.** | **P10** | 0.3191 | **0.2873** | 0.2554 | 0.3875 | 0.2235 | 0.3556 |
| **P03** | **vs.** | **P11** | 0.4068 | **0.3888** | 0.3584 | 0.4542 | 0.3405 | 0.4363 |
| **P03** | **vs.** | **P12** | 0.3938 | **0.365** | 0.3275 | 0.4641 | 0.2987 | 0.4353 |
| **P03** | **vs.** | **P13** | 0.3325 | **0.3122** | 0.2834 | 0.3857 | 0.2631 | 0.3654 |
| **P03** | **vs.** | **P14** | 0.3748 | **0.3467** | 0.33 | 0.4225 | 0.3019 | 0.3945 |
| **P03** | **vs.** | **P15** | 0.3419 | **0.3229** | 0.2902 | 0.3956 | 0.2711 | 0.3765 |
| **P03** | **vs.** | **P16** | 0.3227 | **0.296** | 0.2678 | 0.3858 | 0.2412 | 0.3591 |
| **P03** | **vs.** | **P17** | 0.4084 | **0.3911** | 0.3601 | 0.4614 | 0.3428 | 0.4441 |
| **P03** | **vs.** | **P18** | 0.3175 | **0.2945** | 0.2703 | 0.3685 | 0.2473 | 0.3455 |
| **P04** | **vs.** | **P05** | 0.353 | **0.3239** | 0.2999 | 0.4101 | 0.2709 | 0.381 |
| **P04** | **vs.** | **P06** | 0.3359 | **0.3145** | 0.2922 | 0.3829 | 0.2709 | 0.3615 |
| **P04** | **vs.** | **P07** | 0.4001 | **0.3701** | 0.3361 | 0.4695 | 0.3061 | 0.4395 |
| **P04** | **vs.** | **P09** | 0.4031 | **0.3846** | 0.3588 | 0.45 | 0.3403 | 0.4315 |
| **P04** | **vs.** | **P10** | 0.3348 | **0.2996** | 0.2731 | 0.4031 | 0.2379 | 0.3678 |
| **P04** | **vs.** | **P11** | 0.3836 | **0.3624** | 0.3389 | 0.4371 | 0.3177 | 0.4158 |
| **P04** | **vs.** | **P12** | 0.4317 | **0.4015** | 0.3636 | 0.5037 | 0.3334 | 0.4735 |
| **P04** | **vs.** | **P13** | 0.4325 | **0.4129** | 0.3806 | 0.4901 | 0.361 | 0.4704 |
| **P04** | **vs.** | **P14** | 0.4473 | **0.4189** | 0.3837 | 0.5114 | 0.3553 | 0.4831 |
| **P04** | **vs.** | **P15** | 0.3957 | **0.373** | 0.3431 | 0.4503 | 0.3204 | 0.4277 |
| **P04** | **vs.** | **P16** | 0.3776 | **0.3476** | 0.3205 | 0.4365 | 0.2905 | 0.4065 |
| **comparison** | | | **Mean G’st** | **BC**  **Mean G’st** | **Lower**  **95%CI** | **Upper**  **95%CI** | **BC Lower**  **95%CI** | **BC Upper 95%CI** |
| **P04** | **vs.** | **P17** | 0.3976 | **0.3754** | 0.3439 | 0.4538 | 0.3217 | 0.4316 |
| **P04** | **vs.** | **P18** | 0.3518 | **0.3267** | 0.2991 | 0.4041 | 0.274 | 0.3791 |
| **P05** | **vs.** | **P06** | 0.2916 | **0.2652** | 0.2475 | 0.3416 | 0.221 | 0.3151 |
| **P05** | **vs.** | **P07** | 0.4591 | **0.4287** | 0.4053 | 0.5185 | 0.3749 | 0.4881 |
| **P05** | **vs.** | **P09** | 0.3769 | **0.3542** | 0.3243 | 0.4265 | 0.3016 | 0.4038 |
| **P05** | **vs.** | **P10** | 0.3187 | **0.2799** | 0.2637 | 0.382 | 0.225 | 0.3433 |
| **P05** | **vs.** | **P11** | 0.3728 | **0.348** | 0.3263 | 0.4212 | 0.3015 | 0.3963 |
| **P05** | **vs.** | **P12** | 0.4104 | **0.3775** | 0.3497 | 0.4755 | 0.3169 | 0.4427 |
| **P05** | **vs.** | **P13** | 0.3009 | **0.2737** | 0.2484 | 0.3583 | 0.2211 | 0.3311 |
| **P05** | **vs.** | **P14** | 0.2994 | **0.2605** | 0.2465 | 0.3616 | 0.2076 | 0.3227 |
| **P05** | **vs.** | **P15** | 0.3221 | **0.2934** | 0.275 | 0.3741 | 0.2464 | 0.3454 |
| **P05** | **vs.** | **P16** | 0.3111 | **0.2761** | 0.2626 | 0.3644 | 0.2276 | 0.3294 |
| **P05** | **vs.** | **P17** | 0.3517 | **0.3247** | 0.2997 | 0.4062 | 0.2726 | 0.3791 |
| **P05** | **vs.** | **P18** | 0.2732 | **0.2405** | 0.2245 | 0.3227 | 0.1918 | 0.2901 |
| **P06** | **vs.** | **P07** | 0.2501 | **0.2187** | 0.2016 | 0.3048 | 0.1702 | 0.2733 |
| **P06** | **vs.** | **P09** | 0.3856 | **0.3726** | 0.3351 | 0.4426 | 0.3221 | 0.4297 |
| **P06** | **vs.** | **P10** | 0.2757 | **0.2431** | 0.2184 | 0.3373 | 0.1859 | 0.3048 |
| **P06** | **vs.** | **P11** | 0.312 | **0.2936** | 0.2678 | 0.3567 | 0.2494 | 0.3383 |
| **P06** | **vs.** | **P12** | 0.3348 | **0.305** | 0.2791 | 0.3942 | 0.2493 | 0.3644 |
| **P06** | **vs.** | **P13** | 0.3572 | **0.3392** | 0.311 | 0.403 | 0.293 | 0.385 |
| **P06** | **vs.** | **P14** | 0.3429 | **0.3153** | 0.29 | 0.3987 | 0.2624 | 0.3712 |
| **P06** | **vs.** | **P15** | 0.3623 | **0.3436** | 0.3109 | 0.4134 | 0.2922 | 0.3947 |
| **P06** | **vs.** | **P16** | 0.3847 | **0.3623** | 0.3195 | 0.4471 | 0.2971 | 0.4247 |
| **P06** | **vs.** | **P17** | 0.2635 | **0.2404** | 0.2249 | 0.3084 | 0.2018 | 0.2852 |
| **P06** | **vs.** | **P18** | 0.2901 | **0.2663** | 0.2453 | 0.3399 | 0.2216 | 0.3162 |
| **P07** | **vs.** | **P09** | 0.4388 | **0.4185** | 0.3827 | 0.4986 | 0.3624 | 0.4783 |
| **P07** | **vs.** | **P10** | 0.3278 | **0.2858** | 0.2687 | 0.391 | 0.2268 | 0.349 |
| **P07** | **vs.** | **P11** | 0.36 | **0.331** | 0.2917 | 0.4267 | 0.2627 | 0.3977 |
| **P07** | **vs.** | **P12** | 0.3619 | **0.3204** | 0.2697 | 0.4515 | 0.2283 | 0.41 |
| **P07** | **vs.** | **P13** | 0.439 | **0.4132** | 0.3852 | 0.4976 | 0.3594 | 0.4718 |
| **P07** | **vs.** | **P14** | 0.454 | **0.421** | 0.4046 | 0.5054 | 0.3716 | 0.4725 |
| **P07** | **vs.** | **P15** | 0.4657 | **0.4401** | 0.413 | 0.5228 | 0.3874 | 0.4972 |
| **P07** | **vs.** | **P16** | 0.5675 | **0.5446** | 0.5016 | 0.6341 | 0.4787 | 0.6113 |
| **P07** | **vs.** | **P17** | 0.4238 | **0.3964** | 0.377 | 0.4804 | 0.3496 | 0.453 |
| **P07** | **vs.** | **P18** | 0.3466 | **0.3128** | 0.2894 | 0.4079 | 0.2556 | 0.374 |
| **P09** | **vs.** | **P10** | 0.3905 | **0.3665** | 0.3404 | 0.4475 | 0.3163 | 0.4235 |
| **P09** | **vs.** | **P11** | 0.3977 | **0.3831** | 0.3508 | 0.445 | 0.3362 | 0.4304 |
| **P09** | **vs.** | **P12** | 0.4004 | **0.3749** | 0.3488 | 0.4621 | 0.3234 | 0.4367 |
| **P09** | **vs.** | **P13** | 0.3205 | **0.3024** | 0.2778 | 0.3688 | 0.2597 | 0.3507 |
| **P09** | **vs.** | **P14** | 0.395 | **0.3696** | 0.3425 | 0.4514 | 0.317 | 0.426 |
| **P09** | **vs.** | **P15** | 0.3703 | **0.3516** | 0.3249 | 0.4216 | 0.3061 | 0.4028 |
| **P09** | **vs.** | **P16** | 0.4295 | **0.4097** | 0.3592 | 0.4983 | 0.3394 | 0.4785 |
| **P09** | **vs.** | **P17** | 0.4795 | **0.4652** | 0.4355 | 0.5261 | 0.4212 | 0.5118 |
| **P09** | **vs.** | **P18** | 0.3918 | **0.374** | 0.3445 | 0.4426 | 0.3268 | 0.4248 |
| **P10** | **vs.** | **P11** | 0.3642 | **0.3349** | 0.3079 | 0.4246 | 0.2786 | 0.3953 |
| **P10** | **vs.** | **P12** | 0.319 | **0.2768** | 0.2446 | 0.402 | 0.2024 | 0.3598 |
| **P10** | **vs.** | **P13** | 0.2384 | **0.2027** | 0.1765 | 0.3228 | 0.1408 | 0.2871 |
| **P10** | **vs.** | **P14** | 0.3037 | **0.2609** | 0.2334 | 0.3923 | 0.1906 | 0.3495 |
| **P10** | **vs.** | **P15** | 0.3311 | **0.2988** | 0.2592 | 0.4147 | 0.2269 | 0.3824 |
| **P10** | **vs.** | **P16** | 0.3312 | **0.2916** | 0.2586 | 0.4121 | 0.219 | 0.3725 |
| **P10** | **vs.** | **P17** | 0.3686 | **0.3363** | 0.3098 | 0.438 | 0.2776 | 0.4057 |
| **P10** | **vs.** | **P18** | 0.2659 | **0.2265** | 0.2117 | 0.3309 | 0.1722 | 0.2914 |
| **P11** | **vs.** | **P12** | 0.3266 | **0.2955** | 0.2727 | 0.3879 | 0.2416 | 0.3568 |
| **P11** | **vs.** | **P13** | 0.415 | **0.398** | 0.3669 | 0.4585 | 0.3498 | 0.4414 |
| **P11** | **vs.** | **P14** | 0.4633 | **0.4402** | 0.4159 | 0.5122 | 0.3928 | 0.4891 |
| **P11** | **vs.** | **P15** | 0.4496 | **0.4323** | 0.4032 | 0.4946 | 0.3859 | 0.4773 |
| **P11** | **vs.** | **P16** | 0.5194 | **0.5012** | 0.4603 | 0.5764 | 0.442 | 0.5581 |
| **P11** | **vs.** | **P17** | 0.4374 | **0.4192** | 0.3862 | 0.4844 | 0.368 | 0.4662 |
| **P11** | **vs.** | **P18** | 0.3607 | **0.3394** | 0.3139 | 0.4036 | 0.2925 | 0.3823 |
| **P12** | **vs.** | **P13** | 0.2487 | **0.2123** | 0.1958 | 0.3081 | 0.1595 | 0.2717 |
| **comparison** | | | **Mean G’st** | **BC**  **Mean G’st** | **Lower**  **95%CI** | **Upper**  **95%CI** | **BC Lower**  **95%CI** | **BC Upper 95%CI** |
| **P12** | **vs.** | **P14** | 0.308 | **0.2664** | 0.2443 | 0.3794 | 0.2027 | 0.3379 |
| **P12** | **vs.** | **P15** | 0.3673 | **0.3357** | 0.3089 | 0.4286 | 0.2773 | 0.397 |
| **P12** | **vs.** | **P16** | 0.5128 | **0.4876** | 0.4245 | 0.5977 | 0.3993 | 0.5724 |
| **P12** | **vs.** | **P17** | 0.4753 | **0.4494** | 0.4098 | 0.5451 | 0.3838 | 0.5192 |
| **P12** | **vs.** | **P18** | 0.3819 | **0.3507** | 0.3161 | 0.4478 | 0.2849 | 0.4167 |
| **P13** | **vs.** | **P14** | 0.1661 | **0.1291** | 0.1226 | 0.2205 | 0.0856 | 0.1835 |
| **P13** | **vs.** | **P15** | 0.2185 | **0.1936** | 0.1719 | 0.2656 | 0.1469 | 0.2406 |
| **P13** | **vs.** | **P16** | 0.3378 | **0.3126** | 0.2714 | 0.4103 | 0.2462 | 0.3852 |
| **P13** | **vs.** | **P17** | 0.3771 | **0.3575** | 0.3363 | 0.4259 | 0.3167 | 0.4063 |
| **P13** | **vs.** | **P18** | 0.2578 | **0.2332** | 0.2038 | 0.3156 | 0.1791 | 0.291 |
| **P14** | **vs.** | **P15** | 0.256 | **0.2201** | 0.2036 | 0.3183 | 0.1676 | 0.2824 |
| **P14** | **vs.** | **P16** | 0.2659 | **0.2251** | 0.2055 | 0.3368 | 0.1647 | 0.296 |
| **P14** | **vs.** | **P17** | 0.3895 | **0.3605** | 0.3235 | 0.4608 | 0.2946 | 0.4318 |
| **P14** | **vs.** | **P18** | 0.2945 | **0.2604** | 0.2347 | 0.3652 | 0.2006 | 0.3311 |
| **P15** | **vs.** | **P16** | 0.3686 | **0.3428** | 0.3079 | 0.4379 | 0.282 | 0.4121 |
| **P15** | **vs.** | **P17** | 0.3297 | **0.3069** | 0.2816 | 0.3854 | 0.2588 | 0.3625 |
| **P15** | **vs.** | **P18** | 0.2865 | **0.26** | 0.2311 | 0.344 | 0.2046 | 0.3175 |
| **P16** | **vs.** | **P17** | 0.3811 | **0.3551** | 0.3217 | 0.4514 | 0.2957 | 0.4254 |
| **P16** | **vs.** | **P18** | 0.3319 | **0.3031** | 0.266 | 0.402 | 0.2372 | 0.3732 |
| **P17** | **vs.** | **P18** | 0.2627 | **0.2339** | 0.2101 | 0.3195 | 0.1813 | 0.2907 |

**Table s5** AMOVA results of spatial and temporal variation. Significance level is based on 20,000 permutations.

| *Source of variation* | *df* | *Sum of squares* | *Variance components* | *%*  *variation* | *Fixation*  *indices* | *p* |
| --- | --- | --- | --- | --- | --- | --- |
| Among regions | 2 | 169.260 | 0.096 | 2.01 | FCT 0.020 | p<0.001 |
| Among populations within regions | 14 | 560.452 | 0.549 | 11.48 | FSC 0.117 | p<0.001 |
| Within cohorts | 1137 | 4701.493 | 4.135 | 86.51 |  |  |
| Total | 1153 | 5431.205 | 4.778 |  | FST 0.135 | p<0.001 |

**Table s6** Generalized linear models (GLMs) that fit the variation in expected and observed heterozygosity (He, Ho), inbreeding coefficient (Fis) and allelic richness (Ar) in cohorts 2019 and Soil 2 across populations. The explanatory variables nearest neighbor distance and kettle hole area were z-transformed. Shown are the full models as well as significant reduced models revealed by stepwise backward selection.

| **He 2019** |  |  |  |  |  |
| --- | --- | --- | --- | --- | --- |
|  | *Estimate* | *Std. Error* | *t value* | *p* |  |
| (Intercept) | 0.641246 | 0.01091 | 58.776 | <2e-16 | *** |
| Area | -0.016133 | 0.009602 | -1.68 | 0.1168 |  |
| Nearest Neighbor distance | -0.001741 | 0.007999 | -0.218 | 0.831 |  |
| Area:Nearest Neighbor distance | -0.042938 | 0.023662 | -1.815 | 0.0927 | . |
| Residual deviance: 0.021261 on 13 degrees of freedom |  |  |  |  |  |
| **He Soil2** |  |  |  |  |  |
|  | *Estimate* | *Std. Error* | *t value* | *p* |  |
| (Intercept) | 0.63631 | 0.0192 | 33.149 | 4.70E-07 | *** |
| Area | -0.04103 | 0.03303 | -1.242 | 0.269 |  |
| Nearest Neighbor distance | 0.08018 | 0.0753 | 1.065 | 0.336 |  |
| Area:Nearest Neighbor distance | 0.06946 | 0.11157 | 0.623 | 0.561 |  |
| Residual deviance: 0.0080105 on 5 degrees of freedom |  |  |  |  |  |
| **Ar 2019** |  |  |  |  |  |
|  | *Estimate* | *Std. Error* | *t value* | *p* |  |
| (Intercept) | 4.20625 | 0.09371 | 44.886 | 1.21E-15 | *** |
| Area | -0.08266 | 0.08247 | -1.002 | 0.334 |  |
| Nearest Neighbor distance | -0.11195 | 0.0687 | -1.629 | 0.127 |  |
| Area:Nearest Neighbor distance | -0.21004 | 0.20324 | -1.033 | 0.32 |  |
| Residual deviance: 1.5686 on  13 degrees of freedom |  |  |  |  |  |
| **Ar Soil2** |  |  |  |  |  |
|  | *Estimate* | *Std. Error* | *t value* | *p* |  |
| (Intercept) | 4.6223 | 0.1406 | 32.883 | 4.89E-07 | *** |
| Area | 0.2619 | 0.2419 | 1.083 | 0.328 |  |
| Nearest Neighbor distance | 0.645 | 0.5514 | 1.17 | 0.295 |  |
| Area:Nearest Neighbor distance | 0.7797 | 0.817 | 0.954 | 0.384 |  |
| Residual deviance: 0.42958 on  5 degrees of freedom |  |  |  |  |  |
| **Fis 2019** |  |  |  |  |  |
|  | *Estimate* | *Std. Error* | *t value* | *p* |  |
| (Intercept) | 0.10874 | 0.01946 | 5.589 | 8.79E-05 | *** |
| Area | -0.01685 | 0.01712 | -0.984 | 0.34303 |  |
| Nearest Neighbor distance | 0.04748 | 0.01426 | 3.329 | 0.00544 | ** |
| Area:Nearest Neighbor distance | -0.02574 | 0.0422 | -0.61 | 0.55235 |  |
| Residual deviance: 0.067613 on 13 degrees of freedom |  |  |  |  |  |
| **Fis 2019** |  |  |  |  |  |
|  | *Estimate* | *Std. Error* | *t value* | *p* |  |
| (Intercept) | 0.10332 | 0.01725 | 5.991 | 2.47E-05 | *** |
| Nearest Neighbor distance | 0.04464 | 0.01372 | 3.252 | 0.00536 | ** |
| Residual deviance: 0.074467 on 15 degrees of freedom |  |  |  |  | R^2^*_Pseudo_=*0.456 |
| **Fis Soil2** |  |  |  |  |  |
|  | *Estimate* | *Std. Error* | *t value* | *p* |  |
| (Intercept) | 0.06976 | 0.02292 | 3.043 | 0.0287 | * |
| Area | -0.03728 | 0.03944 | -0.945 | 0.388 |  |
| Nearest Neighbor distance | 0.15729 | 0.08993 | 1.749 | 1.41E-01 |  |
| Area:Nearest Neighbor distance | 0.21999 | 0.13324 | 1.651 | 0.1596 |  |
| Residual deviance: 0.011424 on  5 degrees of freedom |  |  |  |  |  |
| **Ho 2019** |  |  |  |  |  |
|  | *Estimate* | *Std. Error* | *t value* | *p* |  |
| (Intercept) | 0.572884 | 0.016285 | 35.178 | 2.81E-14 | *** |
| Area | -0.001061 | 0.014332 | -0.074 | 0.9421 |  |
| Nearest Neighbor distance | -0.024991 | 0.01194 | -2.093 | 0.0565 | . |
| Area:Nearest Neighbor distance | -0.030108 | 0.03532 | -0.852 | 4.09E-01 |  |
| Residual deviance: 0.047374 on 13 degrees of freedom |  |  |  |  |  |
| **Ho 2019** |  |  |  |  |  |
|  | *Estimate* | *Std. Error* | *t value* | *p* |  |
| (Intercept) | 0.56703 | 0.01414 | 40.111 | <2e-16 | *** |
| Nearest Neighbor distance | -0.02602 | 0.01125 | -2.313 | 0.0353 | * |
| Residual deviance: 0.050034 on 15 degrees of freedom |  |  |  |  | R^2^*_Pseudo_=*0.252 |
| **Ho Soil2** |  |  |  |  |  |
|  | Estimate | Std. Error | t value | p |  |
| (Intercept) | 0.581965 | 0.029201 | 19.93 | 5.88E-06 | *** |
| Area | -0.009825 | 0.050246 | -0.196 | 0.853 |  |
| Nearest Neighbor distance | -0.032595 | 0.114551 | -0.285 | 0.787 |  |
| Area:Nearest Neighbor distance | -0.090943 | 0.169721 | -0.536 | 0.615 |  |
| Residual deviance: 0.018538 on 5 degrees of freedom |  |  |  |  |  |

*Methods*

**Table s7** Details of primers used for microsatellite analyses of Oenanthe aquatica. Name, primer sequence, repeat motif, accession number, annealing temperature (T_m_) and Dye used for multiplexing are given.

| ***Locus name*** | ***Primer sequences (5′–3′)*** | ***Repeat motif*** | ***T_m_ (°C)*** | ***Dye*** | ***Accession no.*** |
| --- | --- | --- | --- | --- | --- |
| O_01 | F: AGTCGCAAGTTAAAGGGAAGC | (AGC)_6_ | 54 | NED | KJ946205 |
|  | R: GGAGCATACTCTTGGGAGAGG |  |  |  |  |
| O_03 | F: AATGTCATTCATCCCACCAC | (TTG)_7_ | 50 | VIC | KJ946207 |
|  | R: TGGGTTCCATGCAAAATTATC |  |  |  |  |
| O_10 | F: ACATCGATCTGGGCTGGTAA | (CA)_11_ | 50 | 6-FAM | KJ946208 |
|  | R: ATTTAATTCAAGGCGTTGCG |  |  |  |  |
| O_13 | F: ACACAAGATTTATAATCTGGCAAA | (AC)_11_ | 53 | NED | KJ946209 |
|  | R: GCAATGACATAGTCCAAGCTG |  |  |  |  |
| O_17 | F:ATCTCAGTGTTATGTGCTGTGTAGA | (TC)_12_ | 52 | VIC | KJ946210 |
|  | R:TCACCGGGCGTTGAATAATA |  |  |  |  |
| O_18 | F: TACACGAAAGCGACGGTGAT | (AG)_12_ | 52 | VIC | KJ946211 |
|  | R: CATCAGGGTCCGATATGACA |  |  |  |  |
| O_20 | F: TTACCGTATTGTTAATTTTACCGGAG | (TC)_12_ | 52 | NED | KJ946212 |
|  | R:TTGCTCGAATTCCAACATAAA |  |  |  |  |
| O_21 | F: CACCCAACAAGAAACAGTACTATAAA | (AC)_12_ | 52 | PET | KJ946213 |
|  | R: TCAAAGCATTCTTGGCCTTC |  |  |  |  |
| O_28 | F: GCACTGTCCCGGTAAGTCTG | (GT)_13_ | 59 | 6-FAM | KJ946215 |
|  | R: AAGTTTGACTGATAAGGTTTCCA |  |  |  |  |
| O_32 | F: AGAAAACGGGGACGAAGAAG | (TG)_13_ | 59 | 6-FAM | KJ946216 |
|  | R: CACCAAGAAGCGACTCCACT |  |  |  |  |
| O_37 | F: TCGATAGCCACAAGAGCAAA | (GA)_15_ | 53 | PET | KJ946218 |
|  | R: TTACAATCATGGCTTCGTGA |  |  |  |  |
| O_38 | F: CAATCCAACACTCTCATTTTCC | (AC)_15_ | 52 | NED | KJ946219 |
|  | R: TCCTAAGCAAAGTCATCAATGC |  |  |  |  |
| O_47 | F: CCATCGATAGCATCCAGGTA | (TG)_18_ | 53 | PET | KJ946220 |
|  | R: AATAGTAATTAGGAATCTCACGCAC |  |  |  |  |
